# Spatiotemporal assessment of protein and lipid oxidation in concentrated oil-in-water emulsions stabilized with legume protein isolates

**DOI:** 10.1016/j.crfs.2024.100817

**Published:** 2024-08-05

**Authors:** Mariska Brüls-Gill, Vincent J.P. Boerkamp, Johannes Hohlbein, John P.M. van Duynhoven

**Affiliations:** a Laboratory of Biophysics, Wageningen University and Research, Stippeneng 4, 6708 WE, Wageningen, the Netherlands; b Laboratory of Self-Organizing Soft Matter, Department of Chemical Engineering and Chemistry & Institute for Complex Molecular Systems, Eindhoven University of Technology, P.O. Box 513, 5600 MB, Eindhoven, the Netherlands; c Laboratory of Food Chemistry, Wageningen University and Research, Bornse Weilanden 9, 6708 WG Wageningen, the Netherlands; d Microspectroscopy Research Facility, Stippeneng 4, 6708 WE Wageningen, the Netherlands; e Unilever Global Foods Innovation Centre, Plantage 14, 6708 WJ Wageningen, the Netherlands

## Abstract

The growing trend of substituting animal-based proteins with plant-based proteins requires more understanding of the functionality and stability of vegan mayonnaises, especially regarding their susceptibility to lipid and protein oxidation. Here, we investigate the spatial and temporal dynamics of lipid and protein oxidation in emulsions stabilized with legume ((hydrolyzed) soy, pea, and faba bean) protein isolates (hSPI, SPI, PPI, FPI). We assessed lipid oxidation globally by NMR and locally by confocal laser scanning microscopy using the oxidation-sensitive fluorescent dye BODIPY 665/676. Further, we assessed local protein oxidation by employing protein autofluorescence and the fluorescently labeled radical spin-trap CAMPO-AFDye 647. Oxidation of oil in droplets was governed by the presence of tocopherols in the oil phase and pro-oxidant transition metals that were introduced via the protein isolates. Non-stripped oil emulsions stabilized with PPI and hSPI displayed higher levels of lipid hydroperoxides as compared to emulsions prepared with SPI and FPI. We attribute this finding to higher availability of catalytically active transition metals in PPI and hSPI. For stripped oil emulsions stabilized with SPI and FPI, lipid hydroperoxide concentrations were negligible in the presence of ascorbic acid, indicating that this agent acted as antioxidant. For the emulsions prepared with PPI and hSPI, lipid hydroperoxide formation was only partly inhibited by ascorbic acid, indicating a role as prooxidant. Interestingly, we observed protein-lipid aggregates in all emulsions. The aggregates underwent fast and extensive co-oxidation, which was also modulated by transition metals and tocopherols originating from the oil phase. Our study demonstrates the potential of spatiotemporal imaging techniques to enhance our understanding of the oxidation processes in emulsions stabilized with plant proteins.

## Highlights

• O/W emulsions were prepared with characterized legume protein isolates.
• Lipid and protein oxidation were assessed in a spatiotemporal manner.
• Lipid oxidation in droplets is modulated by tocopherol and transition metals.
• Protein-lipid aggregates in water phase are prone to lipid and protein oxidation.
• Oxidation in these aggregates is also modulated by tocopherol and transition metals.

## Graphical abstract

**Figure:**
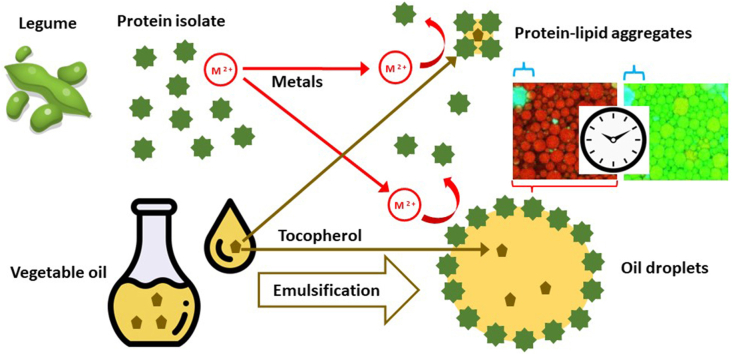

## 1 Introduction

Mayonnaise, a widely consumed sauce, is an oil-in-water emulsion made from egg yolk, vinegar, oil, and spices (Depree and Savage, 2001). By rigorously mixing these ingredients with small amounts of additives, a densely packed emulsion of oil droplets is produced. Egg yolk is a crucial ingredient of mayonnaise, as it contains phospholipids and proteins with emulsifying properties that stabilize the oil droplets. Egg-yolk-based proteins are commonly used in food products due to their excellent stabilizing properties and high solubility in aqueous solutions (Sagis and Yang, 2022). However, the increasing concerns about climate change, population growth, and health issues have led to a growing trend of replacing animal-based proteins with legume proteins, originating from sources such as soy, pea, lentil and faba bean (González-Pérez and Arellano, 2009; Loveday, 2020; Manickavasagan et al., 2022). Although legumes seeds vary substantially in composition, they are all characterized by a high protein content, ranging from 17% to 40%, which makes legumes an attractive source of plant-based proteins (González-Pérez and Arellano, 2009). The main proteins in legumes are albumins and globulins. Albumins have a low molecular weight of about 10–18 kDa and are readily soluble in water. Legume-like globulins are soluble in diluted salt solutions and have considerable higher molecular weight than albumins, typically in the range of 300–370 kDa. Isolation and concentration of proteins involve grinding, oil removal (for lipid rich legumes), protein dissolution in alkali solution, and precipitation under acidic conditions, which strongly modulates protein functionality (Sagis and Yang, 2022) such as emulsifying capacity. Proteins can also undergo partial oxidation during fractionation (Duque-Estrada et al., 2020). It has also been shown that subjecting legume protein isolates to high shear rates as during emulsification, can further promote protein aggregation (Yang et al., 2018). This was confirmed by Keuleyan et al. who observed aggregates of incompletely hydrated protein and lipids (Keuleyan et al., 2023) upon high shear treatment.

Protein-stabilized emulsions like mayonnaise are susceptible to both lipid and protein oxidation, which are closely linked to one another (Yang et al., 2023). Transition metals, such as iron, play a central role since they are potent catalysts for the radical formation of hydroperoxides. These radicals can then generate lipid radicals that consume oxygen in their reaction to hydroperoxides. In this chain reaction, the formation of hydroperoxides is propagated by the redox cycling of transition metals introduced by protein at the droplet interface. Non-adsorbed proteins in the continuous phase can act as antioxidants (Berton et al., 2011) by their ability to scavenge radicals. Proteins can also act as antioxidants by other pathways, including deactivation of reactive oxygen species, binding of pro-oxidative transition metals, reduction of hydroperoxides, and modification of the physical characteristics of the emulsion (Elias et al., 2008). In addition, protein isolates of legumes contain significant amounts of unsaturated fatty acids (González-Pérez and Arellano, 2009; Keuleyan et al., 2023), polyphenols (Wen et al., 2022), polar lipids (Keuleyan et al., 2023), phytic acid (Wang and Guo, 2021) and transition metals (Mesallam et al., 1987), referred to as “co-passengers”, which can have both pro-oxidant and antioxidant effects. Such co-passengers are for example tocopherols, lipid-soluble antioxidants found in concentrations of up to 800 μg/g in lipids, present in pea protein isolate (Keuleyan et al., 2023). Other co-passengers include phospholipids, which can act as antioxidant by replenishing tocopherols at the O/W interface (Cui and Decker, 2016). In addition, phospholipids have been reported to reduce oxidation by binding transition metals (Yoshida et al., 1991). However, the presence of unsaturated bonds, together with a negatively charged headgroup that attracts prooxidant transition metals, renders phospholipids also prone to oxidative reactions (Min and Ahn, 2005). Interestingly, there are also conflicting findings in the literature on instances where phospholipids exhibited no (Yoon and Min, 1987) or even antioxidant effects (Cardenia et al., 2011), demonstrating that the role of phospholipids in oxidation is highly context-dependent.

Although oxidation in vegan mayonnaises has qualitatively been described (Hinderink et al., 2021), quantitative and mechanistic information on multiscale spatial aspects is still limited. Gumus et al. examined the stability of emulsions prepared with lentil, pea, and faba protein isolates against lipid oxidation (Gumus et al., 2017). The authors observed that the presence of non-adsorbed proteins inhibited lipid oxidation. They hypothesized that this inhibition can be attributed to the ability of these proteins to scavenge metal ions, effectively preventing their accumulation at the water-oil interface. Remarkably, Berton et al. observed that in whey protein stabilized emulsions absorbed proteins undergo extensive modifications due to oxidation (Berton et al., 2012). Nevertheless, the unabsorbed proteins significantly slowed down oxidation, which the authors attributed to quenching of free radicals and binding to oxidation initiators like transition metals. Several studies described the use of natural extracts for impeding lipid oxidation in vegan mayonnaises (De Bruno et al., 2021; De Leonardis et al., 2022; Vieira et al., 2023; Włodarczyk et al., 2023). In these studies, antioxidant efficacy was attributed to radical scavenging by water soluble phenolic compounds. This finding stands in contrast to a recent study (Münch et al., 2024) where phenolics that were present as co-passengers in soy and pea protein isolates appeared to act as prooxidants in oil-in-water emulsions, which can be attributed to redox cycling by transition metals (Zhou and Elias, 2012). Another possibility to explain aforementioned conflicting outcomes may lie in the spatial location of the lipid and protein oxidation events. Yan et al. studied the effect of anti- and pro-oxidant co-surfactants on the oxidative stability of modified soy protein, and used microscopy to locate the adsorbed and non-adsorbed protein (Yan et al., 2022). To increase the understanding of the spatial heterogeneity of lipid and protein oxidation within emulsions stabilized with egg yolk and whey protein isolate, Yang et al. employed the hydrophobic oxidation-sensitive fluorophore BODIPY 665/676 combined with autofluorescence of oxidized proteins, originating from reaction of tryptophan with lipid peroxidation products (Yang et al., 2020, 2023). Here, we apply these methodologies to study emulsions stabilized with legume protein isolates. These emulsions will be prepared with soybean oil as is, and after stripping of tocopherols as the dominant oil-soluble antioxidant. We utilize confocal laser scanning microscopy (CLSM) and the oxidation-sensitive fluorescent dye BODIPY 665/676 to track the accumulation of lipid oxidation in emulsions prepared with (hydrolyzed) soy, pea and faba protein isolates, which will be further denoted as hSPI, SPI, PPI and FPI. Protein oxidation is studied using autofluorescence and the spin-trap CAMPO-AFDye 647 to assess local oxidation of proteins in the emulsions. The oxidation is followed over time and the effects of formulating emulsions with different legume protein isolates are quantitatively compared.

## 2 Materials and methods

### 2.1 Materials

Protein isolates from pea, faba bean, and soy were purchased from Pulsin Ltd. (Gloucester, UK). Partially hydrolyzed soy protein isolate (Profam 781) was obtained from ADM (Rolle, Switzerland). Spirit white vinegar (12%) was obtained from Kuhne (Hamburg, Germany) and soybean oil from van der Steen (Vught, The Netherlands). BODIPY 665/676, L-ascorbic acid, and alumina power (Alumina N—Super I) were obtained from Thermo Fischer (Waltham, MA, USA), Aldrich-Europe (Darmstadt, Germany) and MP EcoChrom™ (Eschwege, Germany), respectively. Sodium chloride (>99.5%, EMSURE®), Rhodamine B and EDTA (Ethylenediaminetetraacetic acid disodium salt dihydrate), methanol (≥99.9%, EMSURE® ACS, ISO, Reag. Ph. Eur. for analysis), Folin & Ciocalteu's phenol reagent, gallic acid (97.5–102.5%, titration), and sodium carbonate (anhydrous for analysis EMSURE® ISO) were purchased from Merck. 2-((1*E*,3*E*)-5-((*E*)-3,3-dimethyl-5-sulfo-1-(3-sulfopropyl)indolin-2-ylidene)penta-1,3-dien-1-yl)-3-methyl-1-(6-((4-(2-methyl-1-oxido-3,4-dihydro-2H-pyrrole-2-carboxamido)butyl)amino)-6-oxohexyl)-3-(4-sulfobutyl)-3H-indol-1-ium-5-sulfonate (CAMPO-AFDye 647) was synthesized by SyMO-Chem B.V. (Eindhoven, the Netherlands). The molecular structure of CAMPO-AFDye 647 is shown in the supplementary information (Fig. S1). Deuterated chloroform (CDCl_3_) with 0.03 % tetramethylsilane (TMS), deuterated dimethylsulfoxide (DMSO‑*d*_6_), and deuterated 4 Å molsieves were purchased from Eurisotop (Saint-Aubin, France). Demineralized water (milliQ) was used for all experiments.

### 2.2 Compositional analysis of legume protein isolates

Protein content was determined on dry weight using the Dumas method, using a uniform conversion factor for soy proteins of N = 5,7 (Mariotti et al., 2008, Münch et al., 2024). The degree of hydrolysis (DH) was determined by an established method (Kim et al., 1990). First, the amount of soluble nitrogen (soluble N) was measured in supernatant obtained by mixing 10 ml of inactivated hydrolysate with 10 ml of 20% TCA and then centrifuged at 17000 g for 30 min (Sigma 3-18 KS, Sigma, Ostenrode am Harz, Germany). Then total nitrogen (total N) was determined from 10 ml suspension prepared in the same way and DH is calculated as soluble N/total N*100%. Protein solubility was determined by dispersing 1% of protein isolate in water, followed by centrifugation at 4000 g for 30 min and determining amount of dissolved protein in the supernatant with the Bradford assay. Elemental compositional analysis of the protein isolates was performed by ICP-OES. For assessment of lipids, 1 g of protein isolate was dispersed in demi water in a 50 ml Falcon tube. For assessment of free fatty acids (FFA), monoacylglycerols (MAG), diacylglycerols (DAG), and triacylglycerols (TAG) a threefold volume of 3:1 hexane:isopropanol extraction solvent was added. The tubes were tumbled for 30–45 min and then centrifuged at 4,200 g for 10 min (Sigma 3-18 KS, Sigma, Ostenrode am Harz, Germany). The top layer was separated, put in a 50 ml Falcon tube, and then dried under nitrogen gas flow at 30–35 °C. For assessment of phosphatidylcholine (PC), 2:1 dichloromethane: methanol was added, the tube was also tumbled and the lower layer was taken. All extracts were dried under nitrogen gas flow at 30–35 °C, dried material was dissolved in 1.5 ml 2:1 CDCl_3_:MeOD by vigorous mixing on a thermomixer. Subsequently, 570 μl of the samples was put in 5 mm NMR tubes. The NMR samples were measured at 295 K at 700 MHz on a Bruker Avance HDIII spectrometer under quantitative acquisition conditions. Signals specific for FFA, MAG, DAG and TAG (Hatzakis et al., 2011) were integrated and quantified using PULCON, using 1,2,4,5-tetrachloro-3-nitrobenzene (TCMB) as an external standard. Phytic acid and phospholipids were determined by ^31^P NMR (Boerkamp et al., 2024). In short, the protein isolates were dispersed in a buffer (pH 7.5) solution containing 10 % D_2_O with 120 g/L sodium cholate hydrate, 10 g/L disodium EDTA hydrate, 0.25 g/L trimetaphosphate, and 10 g/L TRIS. This suspension was centrifuged, and the supernatant was analysed by ^31^P NMR. Two independent samples were prepared for each isolate. ^31^P NMR spectra were recorded on a 700 MHz (16.4 T) Bruker Avance III HD NMR spectrometer (Bruker BioSpin, Switzerland) under quantitative conditions. Phytic acid and phospholipids were quantified by using trimetaphosphate as an internal standard.

### 2.3 Extraction of phenolic compounds

Samples of SPI, PPI, FPI or hSPI underwent a two-step extraction process as outlined previously (Alu'datt et al., 2013). Initially, 25 ml of methanol was added to 1 g of each sample and the dispersions were mixed at room temperature for 1 h, followed by centrifugation at 4000 g for 10 min (Sigma Table-Centrifuge 4–10, Ostenrode am Harz, Germany). The residue left from this extraction was subjected to a second extraction with 25 ml of methanol at 60 °C for 1 h, and subsequently centrifuged at 4000 g for 10 min. The resulting supernatant from both extractions was combined and subjected to an additional centrifugation step to eliminate any remaining suspended solids (4000 g, 10 min).

### 2.4 Determination of total phenolic content with Folin-Ciocalteu method

The total phenolic content in each extract was assessed using the Folin–Ciocalteu spectrophotometric method, as originally reported by Singleton and Rossi in 1965 (Singleton and Rossi, 1965), with specific modifications. A standard curve was established by employing a gallic acid stock solution (0.5 mg/ml), ranging from 25 to 87.5 μg/ml. Initially, the undiluted protein extracts were measured. If the absorbance exceeded unity, a 10x dilution in methanol was performed and measured accordingly. To conduct the analysis, 1 ml of the extracts containing phenolic compounds was diluted with 5 ml of distilled water. Subsequently, 0.5 ml of the Folin–Ciocalteu reagent was added to the mixture, followed by 1 ml of saturated sodium carbonate (Na_2_CO_3_) solution. The samples were thoroughly mixed and stored in a dark environment at room temperature. After 1 h, the absorbance was measured at 725 nm to determine the total phenolic content.

### 2.5 Emulsion preparation

Alumina powder was utilized to strip soybean oil of its lipid-soluble antioxidants. The oil and powder were combined at a volume ratio of 1:2 in Falcon tubes, followed by agitation in darkness for 24 h. Subsequently, the mixture underwent centrifugation at 2000 g for 20 min (Sigma Table-Centrifuge 4–10, Ostenrode am Harz, Germany) to separate the stripped soybean oil. The isolated oil was collected, and the centrifugation process was repeated to ensure complete removal of any remaining alumina powder. Stripping efficiency was determined by quantitative comparison of the concentrations of tocopherols before and after stripping. The emulsions were prepared with 60 % (w/w) soybean oil, 1.8 % (w/w) legume protein isolate, 0.76 % (w/w) salt, 35.93 % (w/w) demi water and 1.51 % (w/w) spirit vinegar. For the CLSM experiments, BODIPY 665/676 was then dissolved in either soybean oil or the stripped soybean oil, achieving a final concentration of 1 μM, a level too low to function as an antioxidant (Li et al., 2020). The protein isolate, salt, and demi water were mixed overnight at 300 rpm. Directly before preparing the emulsion, this mixture was agitated at 2000 rpm for 20 s using a Silverson Mixer (East Longmeadow, MA, US). Following this, soybean oil, either unstripped or stripped, was gradually added, and mixed at 8000 rpm for 4 min. Then, spirit vinegar was introduced, and the mixing was continued for another 2 min at 8500 rpm. The pH values of the produced emulsions were 4.3, 4.1, 4.1 and 4.2 for the emulsions prepared with SPI, PPI, FPI and hSPI, correspondingly. When EDTA and ascorbic acid were supplemented, this was done after preparation of the emulsion by gently stirring respectively 1.5 M and 0.15 M solutions into the emulsion, resulting in final concentrations of 10 and 1 mM.

### 2.6 Confocal laser scanning microscopy (CLSM)

#### 2.6.1 Localization of lipid oxidation with BODIPY 665/676

The procedure for monitoring oxidation using CLSM was executed following the methodology outlined by Yang et al. (2020). Individual emulsions (200 μl each) were added into separate wells of μ-slide chambers and maintained at 30 °C to accelerate oxidation. To enable consistent observation of lipid droplets at a specific location throughout the experiment, we etched a cross pattern onto the base of a glass sample carrier (μ-slide 8-well glass bottom, Ibidi®, Munich, Germany) using a diamond knife. Subsequently, the carrier was subjected to a plasma cleaning process (lasting 1 min) to eliminate organic impurities from the glass surface and to prevent adhesion of oil droplets. Once the emulsions were positioned within the carrier, a glass lid was securely placed over it to prevent evaporation. The prepared carrier was then situated on a confocal laser scanning microscope (CLSM, Leica SP8, Wetzlar, Germany) in a manner that allowed revisiting the same position on successive days using the cross-shaped marker. The confocal laser scanning microscope was equipped with a 63x NA = 1.2 water-immersion objective (HC PLAPO CS2, Leica, Wetzlar, Germany), along with a white-light laser featuring selectable excitation wavelengths. The scanning configuration was set to 1200 x 1200 pixels (388 μm by 388 μm), and the line-scanning speed was configured to 600 Hz. Image acquisition was conducted with 6x line-averaging to reduce noise. For the detection of oxidized lipids using BODIPY 665/676 (with a detection range spanning from 580 to 660 nm), the excitation wavelength was set at 561 nm. For measuring non-oxidized lipids, the excitation wavelength was set at 640 nm (with a detection range from 660 to 750 nm). To capture indications of protein oxidation, samples were excited at 488 nm, and fluorescence emission was collected within the range of 500–560 nm. Over a span of 14 days, images were captured at intervals of 2 days.

#### 2.6.2 Localization of free protein radicals with CAMPO-AFDye 647 conjugate

Aliquots of 200 mg of the emulsions prepared as described in section 2.5 were stored in the dark at 30 °C to accelerate oxidation. Prior to imaging, the emulsion was diluted by factor 2 using a mixture of salt, vinegar and milliQ at the same composition as was used to prepare the emulsion water phase. Subsequently, CAMPO-AFDye 647 and Rhodamine B were gently mixed into the diluted emulsion to a final concentration of 1 μM for both. During image acquisition with CLSM, the same settings were used as described in 2.6.1, with the following adjustments. The scanning configuration was set to 512 x 512 pixels (92 μm by 92 μm). For the detection of protein free radicals using CAMPO-AFDye 647 (with a detection range spanning from 660 to 750 nm), the excitation wavelength was set at 640 nm. For measuring all protein with Rhodamine B as general staining agent, the excitation wavelength was set at 561 nm (with a detection range from 580 to 650 nm). Images were recorded consecutively.

### 2.7 Image analysis

For every image, the droplets and aggregates in the waterphase were identified using the 640 nm channel (non-oxidized BODIPY 665/676) and 488 nm channel (protein autofluorescence), respectively. With the ImageJ/Fiji (Schindelin et al., 2012) plugin StarDist (Schmidt et al., 2018), a segmentation process was performed with the low and high percentile values set to 1 and 99.8, respectively. The score threshold was taken as 0.5, alongside an overlap threshold of 0.45. The neural network prediction utilized the adaptable “fluorescence nuclei” model. Employing a custom Python script developed in-house, an analysis was conducted on the distinct droplets recognized by StarDist. The script computes the intersecting area of the droplets, and the mean intensity derived from both non-oxidized and oxidized BODIPY 665/676. For the aggregates, the average intensity of the protein autofluorescence at *λ*_ex 488 nm_ for every individual aggregate was calculated, as well as the aggregate intersecting area.

### 2.8 Quantitative NMR assessment of primary and secondary lipid oxidation products

For the NMR measurements, sets of 5 samples each containing 400 μl of emulsion were prepared in 2 ml Eppendorf tubes and stored at 30 °C. On days 1, 4, 7, 10, and 14, one sample was stored at −80 °C to quench the oxidation reactions. Once all samples were collected, the analysis of the oil phase involved thawing the samples, causing a separation between the oil and water phases. To obtain the oil phases, phase-separated samples were subjected to centrifugation at 2000 g for 5 min (Sigma Table-Centrifuge 4–10, Ostenrode am Harz, Germany). Of each sample, 150 μl of the oil phase was taken out, mixed with 450 μl CDCl_3_:DMSO‑*d*_6_ 5:1 and transferred to NMR tubes. Lipid hydroperoxides and aldehydes were quantified by ^1^H NMR spectroscopy, as previously described (Merkx et al., 2018). In short, single pulse ^1^H and band selective ^1^H spectra were recorded on a 600 MHz (14.1 T) Bruker Neo NMR spectrometer (Bruker BioSpin, Switzerland) equipped with a cryo-probe operating at 295 K. Spectra were apodised using an exponential window function with a 0.1 Hz line broadening, automatically phased, baseline corrected, and integrated in TopSpin v4.1.4 (Bruker BioSpin, Switzerland).

### 2.9 Quantitative NMR assessment of lipids in protein-lipid aggregates

To analyze the lipid content inside the protein-lipid aggregates in the waterphase, emulsions were prepared as described in section 2.5. The emulsions are centrifuged for 1 h at 4000 g (Sigma Table-Centrifuge 4–10, Ostenrode am Harz, Germany), and the pellets are collected, washed with milliQ and freeze-dried. Samples of dried protein-lipid aggregates from the water phase, were extracted in 2:1 dichloromethane:methanol solvent for assessment of FFA, MAG, DAG, TAG and PC using the NMR methods described in section 2.2.

### 2.10 SEM-EDX

Samples of SPI, PPI, FPI and hSPI were individually dispersed in soybean oil, and a small portion of the resulting mixture was applied on to a rivet. Subsequently, the rivet and its attached sample were rapidly frozen using liquid nitrogen. The sample was kept cool and cryoplaned at −110 °C using an ultramicrotome (Leica EM UC7). The prepared samples were transferred to a scanning electron microscope (FIB-SEM Zeiss Auriga) equipped with a Gatan Alto 2500 cyro-stage, cryo-transfer and coating system and sublimated at −90 °C for 5 min. The samples were sputter-coated with platina for 120 s at an angle of 0°. To ensure an even coating, the samples were also sputter-coated with platinum for another 60 s while being gently tilted from −90° to +90°. For imaging purposes, the samples were observed under high vacuum conditions at −125 °C using the (Everhart-Thornley) SE2 and EDX (Oxford X-Max 80 mm) detectors at 10 kV, with a working distance (WD) of 5 mm.

## 3 Results and discussion

### 3.1 Legume protein isolates composition

As previous work demonstrated the ambiguous pro- or antioxidant roles of co-passengers such as transition metal ions and phospholipids (Münch et al., 2024), we performed compositional analyses to assess whether we can relate the concentrations of these co-passengers to the lipid and protein oxidation rates in the emulsions. To assess the composition of the legume protein isolates, we conducted analyses on protein content and co-passengers such as free fatty acids, di- and triacylglycerides, phospholipids, transition metals and phenolic compounds. Furthermore, considering the demonstrated impact of unabsorbed proteins on reducing lipid oxidation in the dispersed oil phase, the protein solubility was measured. The most relevant parameters are summarized in Table 1, additional ones can be found in Table S1. The concentrations of most trace minerals were similar among the investigated legume protein isolates. The most noticeable variations were observed in the levels of iron and calcium. Specifically, PPI comprises approximately three times the iron content compared to SPI and hSPI (5.1 versus 1.6–1.8 mmol/kg). FPI, on the other hand, demonstrated an intermediate iron level of 3.3 mmol/kg, being similar to the iron concentration found in egg yolk (ranging from 2.7 to 3.0 mmol/kg) (Buckiuniene et al., 2016). The isolates also contained considerable amounts of phytic acid, a known chelator of polyvalent cations (Wang and Guo, 2021; Empson et al., 1991; Graf and Eaton, 1990). The ratio of polyvalent cations (Fe, Cu, Ca, Zn, Mn) to phytic acid ranged from 0.6 to 2.6, so all close to the equivalent point. This may render chelation of Fe and Cu cations incomplete and therefore catalytic action of these transition metals may not be fully nullified. By utilizing SEM-EDX, we also identified the presence of small particles containing magnesium, silica, aluminum, and iron (Fig. S2). The free fatty acid (FFA) and diacylgycerol (DAG) contents fall in narrow ranges, respectively 0.2–0.3 and 0.1–0.2 g/100 g. FPI and hSPI contained considerable levels of triacylglycerols, respectively 0.5 and 1.0 g/100 g. We also assessed the content of phospholipids, co-passengers with emulsifying and antioxidant properties. FPI and PPI are higher in phospholipid content (3.2 and 4.5 g/100 g respectively) compared to SPI and hSPI (2.3 g/100 g). Within the protein isolates all (α-, β-, γ-, δ-) tocopherol concentrations were below the limit of detection (<10 mg/kg).

**Table 1:** Characteristics of the legume protein isolates: protein content (N = 5,7), concentrations of free fatty acids (FFA) and diacylglycerols (DAG), triacylglycerols (TAG), total phospholipids (PC, PE and PI), protein solubility, cupper (Cu), iron (Fe), total concentration of polyvalent cations (Fe, Cu, Ca, Zn, Mn), phytic acid and free phenolic compounds. We note that no monoacylglycerol (MAG) was detected in the isolates. The concentrations of the respective phospholipids can be found in Table S1. Protein solubility is determined by centrifuging at 1% dispersion at 4000 g for 30 min and determining fraction of dissolved protein in the supernatant. Data are based on dry weight and presented as mean of duplicates or as mean±SD.

	Protein content	FFA	DAG	TAG	PL	Protein solubility	Cu	Fe	Fe + Cu + Ca + Zn + Mn	Phytic acid	Phytic acid/(Fe + Cu + Ca + Zn + Mn)	Free phenolic
	% (w/w)	g/100 g	g/100 g	g/100 g	g/100 g	%	mmol/kg	mmol/kg	mmol/kg	mmol/kg		mg/kg
**SPI**	83.6	0.2	0.1	0.2	2.3	3.9 ± 0.2	0.2	1.6	25.1	15.2	0.6	1.3
**FPI**	84.4	0.3	0.2	0.5	3.2	16.3 ± 1.4	0.3	3.3	12.7	33.3	2.6	0.7
**PPI**	75.8	0.3	0.1	0.3	4.5	9.1 ± 1.0	0.2	5.1	14.5	21.2	1.5	1.0
**hSPI**	90.6	0.2	0.1	1.0	2.3	21.3 ± 1.2	0.2	1.8	12.5	25.8	2.1	7.0

### 3.2 Kinetics of primary and secondary oil oxidation

**Fig. 1:**
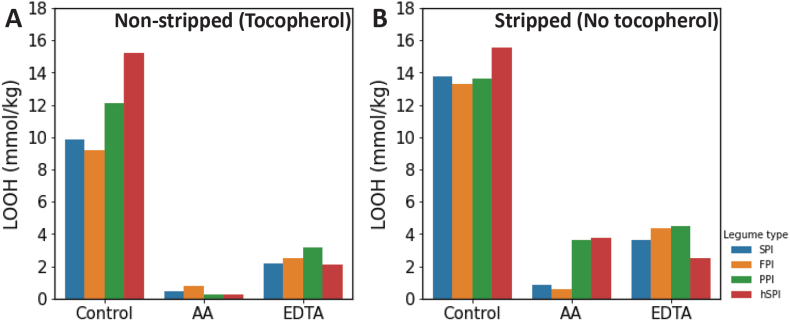
Comparison of the lipid hydroperoxide (LOOH) concentration in emulsions stored at 30 °C for 14 days for emulsions prepared with A) non-stripped oil and B) stripped oil. Emulsions were measured without antioxidants (control) and in the presence of ascorbic acid (AA) and EDTA.

Concentrated oil-in-water emulsions (60% oil) were prepared with the four protein isolates. These emulsions were prepared with spirit vinegar, resulting in pH values in the 4.1–4.3 range. We used quantitative nuclear magnetic resonance (NMR) to assess the accumulation of primary oxidation products (Fig. 1), lipid hydroperoxides (Fig. S3), and secondary oxidation products, aldehydes (Fig. S4), within the oil phase of the emulsions as a function of incubation time. Since we made observations at an early stage of oxidation, we observed a relatively limited production of aldehydes. Hence, we will only discuss effects on hydroperoxide formation. Emulsions prepared with stripped oil, thus with reduced tocopherol levels, exhibited the highest accumulation of lipid hydroperoxides (Fig. 1). In the presence of tocopherols, the hSPI emulsion displayed the most significant lipid hydroperoxide accumulation at day 14 (15 mmol/kg), followed by the PPI emulsion (12 mmol/kg), whereas the SPI and FPI emulsions showed lower accumulation (approximately 10 mmol/kg for both). Upon removal of tocopherols, lipid hydroperoxide accumulation only slightly increased for hSPI and PPI emulsions (to 16 and 14 mmol/kg, respectively), while for the SPI and FPI emulsions, it rose from 10 to 14 mmol/kg. The addition of EDTA effectively reduced lipid hydroperoxide accumulation to values below 5 mmol/kg for all emulsions, indicating that the phytic acid present in the isolates was not fully effective as a chelator of catalytically active transition metals. This can be attributed to the abundance of competing polyvalent cations (Table 1) and/or interactions with proteins (Wang and Guo, 2021).

Notably, ascorbic acid even more strongly decreased lipid hydroperoxide accumulation to below 1 mmol/kg for SPI and FPI emulsions prepared with non-stripped oil. This implies that the antioxidant radical scavenging effect of ascorbic acid is dominant over the known pro-oxidant effect via redox cycling of transition metals. However, for the hSPI and PPI emulsions prepared with stripped oil we observed higher lipid hydroperoxide accumulation in the presence of ascorbic acid (4 mmol/kg). A reason why this trend is not observed for the emulsions prepared with SPI and FPI, could be that in these emulsions transition metals have more affinity to these proteins, which renders them less effective for redox cycling of transition metals.

### 3.3 Spatiotemporal mapping of oxidation of dispersed oil

**Fig. 2:**
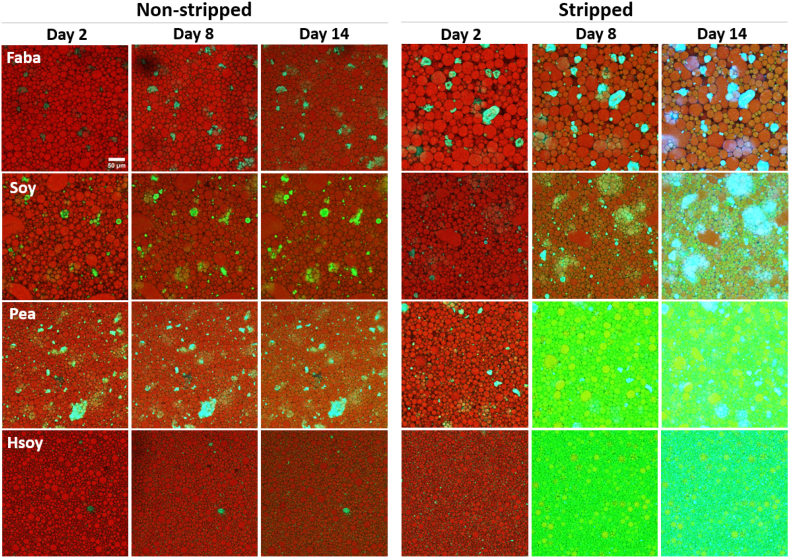
Confocal microscopy images of non-stripped (NS) oil and stripped oil (ST) emulsions after 2, 8 and 14 days of storage at 30 °C. The images are an overlay of the three channels, representing BODIPY 665/676 intensity at λ_ex_ 640 nm/λ_em_ 660-750 nm (non-oxidized lipids) in red, BODIPY 665/676 intensity at λ_ex_ 561 nm/*λ*_em_ 580–660 nm (oxidized lipids) in green and protein autofluorescence at λ_ex_ 488 nm/*λ*_em_ 500–560 nm (oxidized protein) in blue. The emulsions were prepared with 1.8 wt% SPI, FPI, PPI or hSPI. (For interpretation of the references to colour in this figure legend, the reader is referred to the Web version of this article.)

We utilized confocal laser scanning microscopy (CLSM) to track the oxidation of the oil phase within the emulsions over time in a droplet specific manner. To achieve this, we labeled the oil phase with the oxidation-sensitive dye BODIPY 665/676 (Raudsepp et al., 2014a, 2014b) before emulsion preparation. Our findings indicate that in emulsions prepared with non-stripped oil, and therefore in the presence of tocopherols, oxidation within the oil droplets remains minimal across all emulsions (Fig. 2). In line with the NMR result, the addition of EDTA significantly reduced both lipid and protein oxidation in the stripped oil emulsions (Fig. S5). Also, in line with the formation of lipid hydroperoxides measured with NMR, oxidation is more pronounced in emulsions containing PPI and hSPI compared to those with SPI and FPI, with FPI emulsions exhibiting the slowest oxidation rate. In the water phase we observed the presence of oxidized BODIPY 665/676 dye already within the first days. Interestingly, this fluorescence signal originates from aggregates present in the continuous aqueous phase of all emulsions prepared in this study. These aggregates have previously been documented in legume protein isolate based emulsions (Yang et al., 2018). A recent study showed that application of high shear to legume protein isolates can lead to aggregates of proteins and lipids, the latter including phospholipids (Keuleyan et al., 2023). For our legume protein isolate based emulsions, the concentrations of lipids inside the protein-lipid aggregates of the water phase were higher than in the isolates used (Fig. S6 and Table S2). This finding indicates migration of these lipid compounds from the oil phase towards the protein-lipid aggregates in the water phase during emulsification. The addition of ascorbic acid also effectively reduced lipid oxidation in the protein-lipid aggregates in the stripped oil emulsions, although a considerable amount of protein oxidation still occurred (Fig. S7).

### 3.4 Quantitative image analysis of oxidation of dispersed oil

**Fig. 3:**
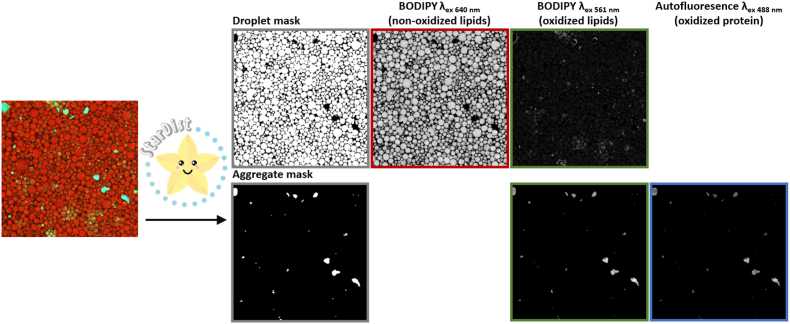
Schematic overview of lipid-oxidation image analysis. The emulsion droplets are imaged using confocal laser scanning microscopy capturing fluorescence intensity from non-oxidized and oxidized BODIPY 665/676. StarDist is used to identify and localize individual droplets and aggregates. Using a tailor-made python script, the fluorescence intensity of non-oxidized and oxidized BODIPY 665/676 in every individual droplet is calculated. Similarly, the fluorescence intensity of oxidized BODIPY 665/676 and protein autofluorescence in every protein aggregate is determined.

**Fig. 4:**
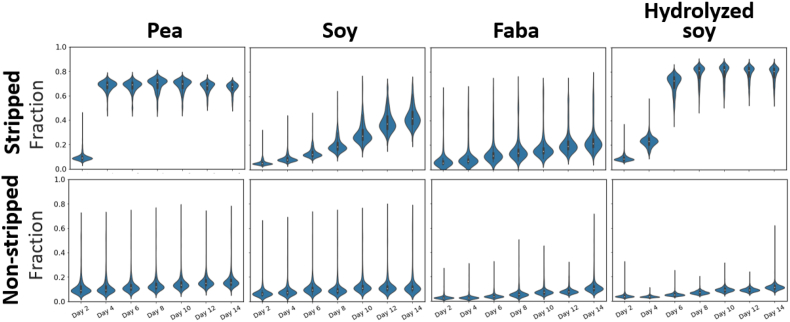
Fraction of BODIPY 665/676 fluorescence intensity at λ_ex_ 561 nm/*λ*_em_ 580–660 nm (oxidized lipids) of the total BODIPY 665/676 fluorescence intensity in the emulsion droplets as a function of day of storage at 30 °C. Emulsions are prepared with non-stripped or stripped oil, and with 1.8 wt% SPI, FPI, PPI or hSPI.

To gain quantitative insights into the oxidation process, we employed image analysis to analyze both protein and lipid oxidation in the emulsions (Fig. 3). To study the lipid oxidation in the dispersed oil phase, we first identified the individual oil droplets. We then compared their average fluorescence intensities and sizes. Fig. 4 illustrates that the fraction of oxidized BODIPY 665/676 of the total BODIPY 665/676 fluorescence intensity, referred to as the oxidation fraction, increases more significantly in stripped emulsions, indicating faster emulsion droplet oxidation in the absence of tocopherols. The oxidation fraction is calculated as the fluorescence intensity detected in the 561 nm channel divided by the sum of the fluorescence intensities detected in the 561 and 640 nm channel. Intriguingly, in non-stripped oil emulsions, oxidation occurs at a much slower rate compared to egg yolk emulsions studied by Yang et al. (2020). While the non-stripped emulsions examined in this study only reach an oxidation fraction of maximally 0.18 at day 14, in the egg yolk emulsion stored at the same temperature the ratio already was 0.32 at day 10. However, in stripped oil emulsions, the oxidation rate varies based on the protein type. For stripped oil SPI and FPI emulsions, oxidation is slower than in egg yolk mayonnaise, whereas for PPI and hSPI emulsions, lipid oxidation occurs notably faster, quickly reaching an oxidation fraction of 0.69 ± 0.05 in four days for PPI, and 0.73 ± 0.08 in six days for hSPI, compared to a steady increase until 0.85 in ten days in egg yolk emulsions. The addition of ascorbic acid or EDTA as water-soluble antioxidants effectively reduces oxidation, with only a slight degree of oxidation observed for the hSPI emulsion with ascorbic acid (Fig. S8).

Among the stripped oil emulsions without water-soluble antioxidants, the lipids in emulsions prepared with PPI and hSPI oxidize faster (oxidation fraction of 0.67 ± 0.04 and 0.82 ± 0.06 at day 14, respectively) than those prepared with SPI or FPI (oxidation fraction of 0.45 ± 0.10 and 0.26 ± 0.14 at day 14, respectively). This observation is in line with the trends in LOOH kinetics measured with NMR as presented in section 3.2. The faster lipid oxidation observed for PPI and hSPI emulsions can only partially be explained by variation of oil droplet size distributions of these emulsions. While the emulsion prepared with FPI had both the slowest oxidation rate and the largest droplet size (Fig. S9), we see differences in the oxidation rates between emulsions with similar droplet sizes, such as those prepared with PPI and SPI. Also, both the PPI and hSPI emulsions had a similarly high oxidation rate, while the hSPI emulsion has smaller average droplet size than the PPI emulsion (3 ± 1 μm vs. 5 ± 2 μm). Protein isolates with lower effective molar mass have a higher diffusion coefficient and can therefore more easily stabilize a newly formed oil-water interface, resulting in smaller droplets (González-Pérez and Arellano, 2009). This is likely the reason why the emulsion prepared with the hydrolyzed soy protein shows the smallest droplet size. Other factors, including the presence of co-passengers such as transition minerals (Duque-Estrada et al., 2020) or polyphenols (Padhi et al., 2017) may be at play. Despite observing a large number of outliers in every image, testing the effect of droplet size on lipid oxidation revealed no significant dependence (Fig. S10), suggesting efficient inter-droplet transport of lipid oxidation intermediates in high-oil emulsions with closely packed droplets (Yang et al., 2020). While polyphenols have been proposed to inhibit lipid peroxidation by acting as radical scavengers (Forman et al., 2014), we observed the highest rate of oxidation for hSPI, which had the highest level of free phenolic compounds. This is in line with a recent study (Münch et al., 2024), in which oil-in-water emulsions prepared with soy and pea protein isolates with high free phenolic levels were also more prone to oxidize.

We note that the lipid hydroperoxide formation levels observed by NMR are in a different range than the oxidation degree measured by BODIPY 665/676. This can be attributed to the shift in BODIPY 665/667 being determined by the time-integral of hydroperoxide radical formation, which is not linearly related to hydroperoxide concentration.

### 3.5 Quantitative image analysis of lipid and protein oxidation in continuous aqueous phase

**Fig. 5:**
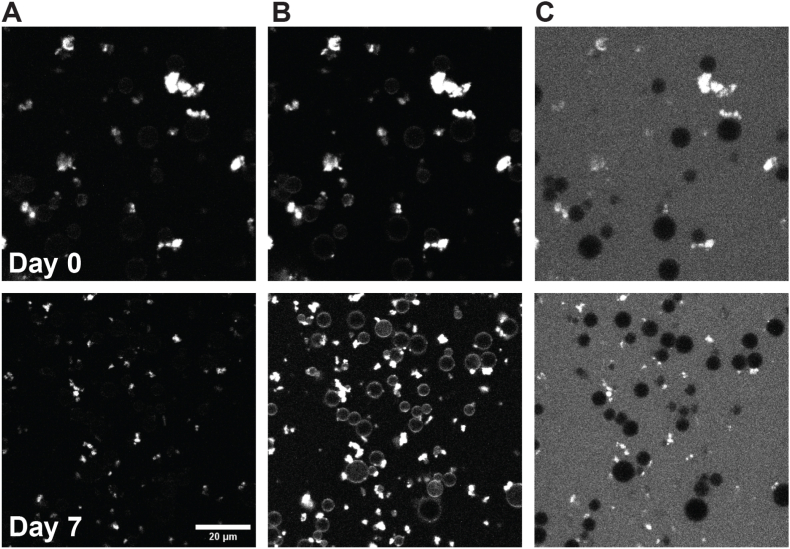
Confocal laser scanning microscopy images of 30% oil emulsion prepared with stripped oil and 1.8 wt% hSPI, before (top row) and after 7 days (bottom row) of storage at 30 °C. A) Protein autofluorescence. B) Protein visualized with general staining agent Rhodamine B. C) Protein free radical spin adduct accumulation visualized with CAMPO-AFDye 647. Images of protein autofluorescence, protein labeled with Rhodamine B and oxidized protein labeled with CAMPO-AFDye 647 are from the same field of view.

To assess protein oxidation in the continuous aqueous phase, we employed the spin-trap CAMPO conjugated to the fluorophore AFDye 647 (CAMPO-AFDye 647). While BODIPY 665/676 reacts with lipid radicals, CAMPO-AFDye 647 forms protein free radical spin adducts. The selectivity of CAMPO-AFDye 647 for oxidized proteins in emulsions, as opposed to proteins in general, was previously confirmed (Yang et al., 2023). Using CAMPO-AFDye 647, we clearly observed occurrence of protein oxidation to a greater extent in the protein-lipid aggregates than at the oil-water interface (Fig. 5). This observation is in line with a higher local concentration of proteins in the protein-lipid aggregates compared to the interface leading to higher local accumulation of the CAMPO-AFDye-647 spintrap.

**Fig. 6:**
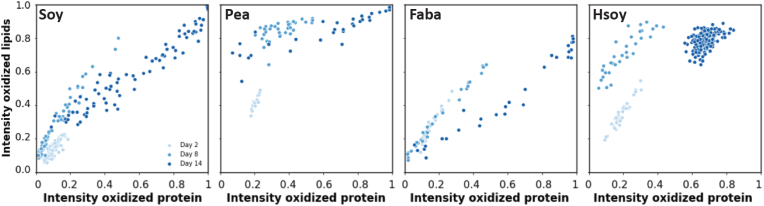
Intensity of BODIPY 665/676 fluorescence at λ_ex_ 561 nm/*λ*_em_ 580–660 nm (oxidized lipids) versus protein autofluorescence at λ_ex_ 488 nm/*λ*_em_ 500–560 nm (oxidized protein). Emulsions are prepared with stripped oil, and with 1.8 wt% SPI, PPI, FPI or hSPI. The emulsions were stored at 30 °C for 14 days. Intensity values were normalized to the same maximum intensity value.

Due to the distinct oxidation behaviors observed for droplets and protein-lipid aggregates, we decided to separately analyze the increase in lipid and protein oxidation. In our approach we monitored peroxyl radical formation by BODIPY 665/676 and protein oxidation by increase in protein autofluorescence. The hSPI emulsions exhibited the smallest protein-lipid aggregates, all measuring smaller than 3 μm^2^ (Fig. S11). In the stripped oil emulsions, we observed that peroxyl radical formation occurred before the increase of protein autofluorescence inside the aggregates for all proteins. The increase in protein autofluorescence was substantial from day 8 to day 14 (Fig. 6). The extent of protein oxidation was similar across all emulsions. Interestingly, ascorbic acid did not reduce protein oxidation (Fig. S12). However, it effectively reduced peroxyl radical formation for PPI and hSPI emulsions as observed by a shift in BODIPY 665/676 fluorescence. The non-stripped emulsions showed negligible protein oxidation (Fig. S13) but significant peroxyl radical formation (Fig. S14), notably higher than observed in the droplets, especially for the SPI and FPI emulsions. This indicates that during emulsification, tocopherols from the non-stripped oil partly migrate into the protein-lipid aggregates, where they act as antioxidants. Whereas the legume isolates themselves did not contain measurable amounts of tocopherols, we measured significant levels of tocopherols in the protein-lipid aggregates of the corresponding emulsions (Fig. S15). The transfer from oil to the protein aggregates is likely facilitated by the affinity of tocopherols for both proteins and phospholipids present in the protein isolates. Transfer of tocopherols towards the aggregates may also be enhanced by micellisation of phospholipids (Barouh et al., 2022).

### 3.6 Potential anti- and pro-oxidant mechanisms at play in legume protein isolate stabilized emulsions

**Fig. 7:**
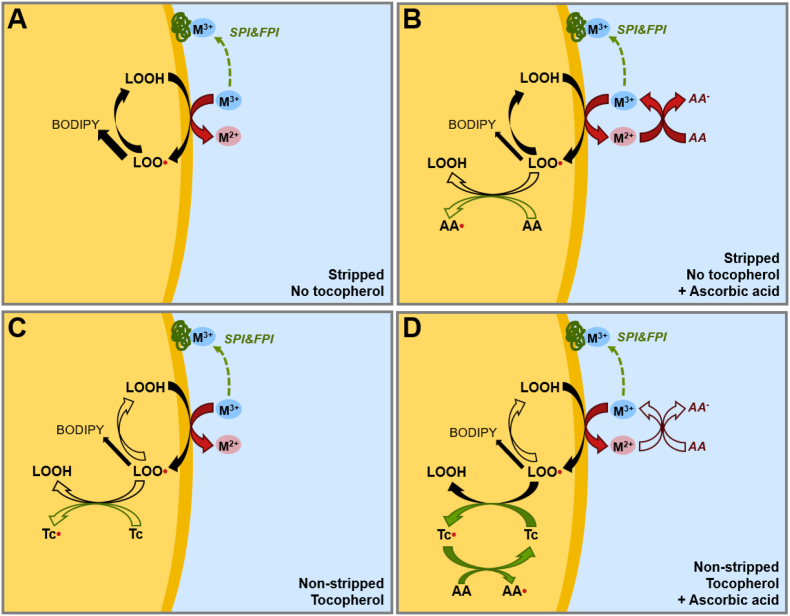
Schematic representation of mechanisms at play in the emulsions prepared with legume protein isolates (A) with and (C) without stripping of the oil resulting in removal of tocopherols (Tc). In B and D, the pathways are depicted when ascorbic acid (AA) is added to the emulsions prepared with respectively stripped and non-stripped oil. Green and red arrows respectively depict anti- and pro-oxidant effects. Filled and closed lines respectively indicate fast and slow reaction rates. (For interpretation of the references to colour in this figure legend, the reader is referred to the Web version of this article.)

Within the schemes presented in Fig. 7 we captured our explanation of the mechanisms at play for the droplet phase in the legume protein isolate based emulsions prepared with stripped and non-stripped oil. We note that these schemes do not attempt to represent the full complexity of oil oxidation in food emulsions (Hennebelle et al., 2024), rather we focus on pathways that are relevant to explain our observations. A critical pathway is the formation of peroxyl radicals (LOO^∙^) from peroxides (LOOH) catalyzed by transition metals. The peroxyl radicals can engage in further reactions that form hydroperoxides, alkoxy radicals and, ultimately, epoxides, volatile aldehydes and polymers (Hennebelle et al., 2024). Fig. 7A shows the situation for stripped oil in absence of tocopherols and ascorbic acid. In that case we observed rapid transition metal catalyzed formation of peroxyl radicals (BODIPY 665/676) and hydroperoxides (NMR). When ascorbic acid was added to the emulsion prepared with stripped oil (Fig. 7B), we observed nearly full inhibition of both peroxyl radical formation and lipid hydroperoxide formation for SPI and FPI emulsion. We attribute this observation to the scavenging of peroxyl radicals by ascorbic acid. For PPI and hSPI emulsions, only partial inhibition is observed, which indicates that besides acting as a radical scavenger, ascorbic acid can also act as pro-oxidant by recycling of transition metals, as is shown in Fig. 7B. Apparently, less transition metals are available for recycling of transition metals in SPI and FPI emulsions by ascorbic acid. This finding cannot be solely explained by iron and copper content in the protein isolates, and neither by their chelation by phytic acid (Table 1). Hence, our current explanation is that the proteins inside SPI and FPI have stronger affinity for these metal ions than PPI and hSPI, thus impeding degradation of hydroperoxides and further lipid and protein oxidation.

For emulsions prepared with non-stripped oil, we observed that the presence of tocopherols result in stronger inhibition of peroxyl radical (BODIPY 665/676) and peroxide (NMR) formation for SPI and FPI compared to FPI and hSPI emulsions. As shown in Fig. 7C, this effect can also be explained by a higher availability of catalytically active transition metals in PPI and hSPI emulsions, their pro-oxidant activity can apparently not be alleviated by the presence of tocopherols. Upon addition of ascorbic acid, we observed strong inhibition of lipid oxidation in all emulsions. This finding is in line with the known synergistic effect of ascorbic acid and tocopherols (Fig. 7D), which overrules any effect of the legume protein isolate used for emulsification. It appears that when both ascorbic acid and tocopherols are present, the radical scavenging function of ascorbic acid is more dominant than its role in redox cycling. We note that addition of EDTA has a strong inhibiting effect on the formation of both hydroperoxides and peroxyl radicals for the oil droplet phase. On the one hand, this inhibiting effect is less effective than the radical scavenging effect of ascorbic acid, in particular when ascorbic acid can act in synergy with tocopherols. On the other hand, the inhibiting effect of EDTA surpasses aforementioned binding of metal ions by proteins inside SPI and FPI. Our results are not conclusive on the interplay of lipid oxidation in the oil droplet and protein-lipid aggregates. To better understand the impact of the protein-lipid aggregates on the oxidation kinetics of the droplet phase, it would be of interest to repeat the spatiotemporal imaging on emulsions in which these protein-lipid aggregates have been removed.

## 4 Conclusion

Our study aimed to localize lipid and protein oxidation in emulsions stabilized with legume protein isolates (hSPI, SPI, PPI, and FPI) using CLSM. We observed that oxidation of oil in the droplet phase is governed by the presence of tocopherols in the oil phase and pro-oxidant transition metals that are introduced as co-passengers. By using NMR, we first established that non-stripped oil emulsions prepared with PPI and hSPI displayed higher levels of lipid hydroperoxides after 14 days of storage as compared to emulsions prepared with SPI and FPI. This difference in oil oxidation kinetics between SPI-, FPI- vs PPI-, hSPI-based emulsions was also semi-quantitatively observed with the fluorescent lipid oxidation marker BODIPY 665/676. We attribute this difference to a higher availability of catalytically active transition metals in PPI and hSPI, due to lower metal chelating activity compared to SPI and FPI. For stripped oil emulsions prepared with SPI and FPI emulsifiers, lipid hydroperoxide concentration after 14 days of storage was negligible when ascorbic acid was supplemented, indicating it acts as a strong antioxidant. In contrast, for the emulsions prepared with PPI and hSPI, lipid hydroperoxide concentrations were only partly reduced in the presence of ascorbic acid. This observation indicates that for PPI and hSPI emulsions ascorbic acid may also act as a prooxidant, likely by redox cycling of available transition metals. Localizing lipid and protein oxidation with BODIDY 665/676, CAMPO-AFDye 647 and protein autofluorescence provided further understanding of the mechanisms at play. We further observed protein-lipid aggregates in the water phase of all emulsions. Both protein autofluorescence and accumulation of the free radical spin-trap CAMPO-AFDye 647 demonstrate extensive protein oxidation occurring inside these aggregates. In addition, lipids in these aggregates co-oxidize with proteins, as observed by a shift in BODIPY 665/676 fluorescence. Lipid oxidation kinetics in the protein-lipid aggregates is substantially faster than those of the droplet phase and is modulated by both tocopherols originating from the oil phase and by transition metals introduced as co-passengers in the legume protein isolates.

## Funding

This work is part of the research programs LocalBioFood and LICENSE (project nos. 731.017.204 and 731.017.301), which are financed by the Dutch Research Council (NWO).

## CRediT authorship contribution statement

**Mariska Brüls-Gill:** Conceptualization, Methodology, Formal analysis, Investigation, Writing – original draft. **Vincent J.P. Boerkamp:** Methodology, Formal analysis, Writing – review & editing. **Johannes Hohlbein:** Conceptualization, Methodology, Validation, Investigation, Writing – review & editing, Project administration, Funding acquisition. **John P.M. van Duynhoven:** Conceptualization, Methodology, Validation, Investigation, Resources, Writing – review & editing, Supervision, Project administration, Funding acquisition, All authors have read and agreed to the published version of the manuscript.

## Declaration of competing interest

The authors declare the following financial interests/personal relationships which may be considered as potential competing interests:

J.P.M. van Duynhoven reports financial support was provided by 10.13039/100007190Unilever Global Innovation Centre Wageningen (The Netherlands). J.P.M. van Duynhoven reports a relationship with Unilever Global Food Innovation Centre that includes: employment. If there are other authors, they declare that they have no known competing financial interests or personal relationships that could have appeared to influence the work reported in this paper.

## Data Availability

Data will be made available on request.
