# Supplementary material for: Spatiotemporal assessment of protein and lipid oxidation in concentrated oil-in-water emulsions stabilized with legume protein isolates
Source: Curr Res Food Sci. 2024 Aug 5;9:100817. doi: 10.1016/j.crfs.2024.100817 (PMC11369386; doi:10.1016/j.crfs.2024.100817)
Supplement: Multimedia component 1 [file mmc1.docx]

Supplementary Information

**Spatiotemporal assessment of protein and lipid oxidation in concentrated oil-in-water emulsions stabilized with legume protein isolates**

Mariska Brüls-Gill^1,2^, Vincent Boerkamp^3^, Johannes Hohlbein^1,4^, John P. M. van Duynhoven^1,5^

^1^ Laboratory of Biophysics, Wageningen University and Research, Stippeneng 4, 6708 WE Wageningen, the Netherlands

^2^ Laboratory of Self-Organizing Soft Matter, Department of Chemical Engineering and Chemistry & Institute for Complex Molecular Systems, Eindhoven University of Technology, P.O. Box 513, 5600 MB, Eindhoven, the Netherlands

^3^Laboratory of Food Chemistry, Wageningen University and Research,  Bornse Weilanden 9, 6708 WG Wageningen, the Netherlands

^4^ Microspectroscopy Research Facility, Stippeneng 4, 6708 WE Wageningen, the Netherlands^5^ Unilever Global Foods Innovation Centre, Plantage 14, 6708 WJ Wageningen, the Netherlands

Content

[Supplementary Figure S1. 3](#_Toc167271070)

[Supplementary Figure S2. 4](#_Toc167271071)

[Supplementary Figure S3. 5](#_Toc167271072)

[Supplementary Figure S4 6](#_Toc167271073)

[Supplementary Figure S5. 7](#_Toc167271074)

[Supplementary Figure S6. 8](#_Toc167271075)

[Supplementary Figure S7. 9](#_Toc167271076)

[Supplementary Figure S8. 10](#_Toc167271077)

[Supplementary Figure S9. 11](#_Toc167271078)

[Supplementary Figure S10. 12](#_Toc167271079)

[Supplementary Figure S11. 13](#_Toc167271080)

[Supplementary Figure S12. 14](#_Toc167271081)

[Supplementary Figure S13. 15](#_Toc167271082)

[Supplementary Figure S14 16](#_Toc167271083)

[Supplementary Figure S15. 17](#_Toc167271084)

[Supplementary Table S1. 18](#_Toc167271085)

[Supplementary Table S2. 19](#_Toc167271086)


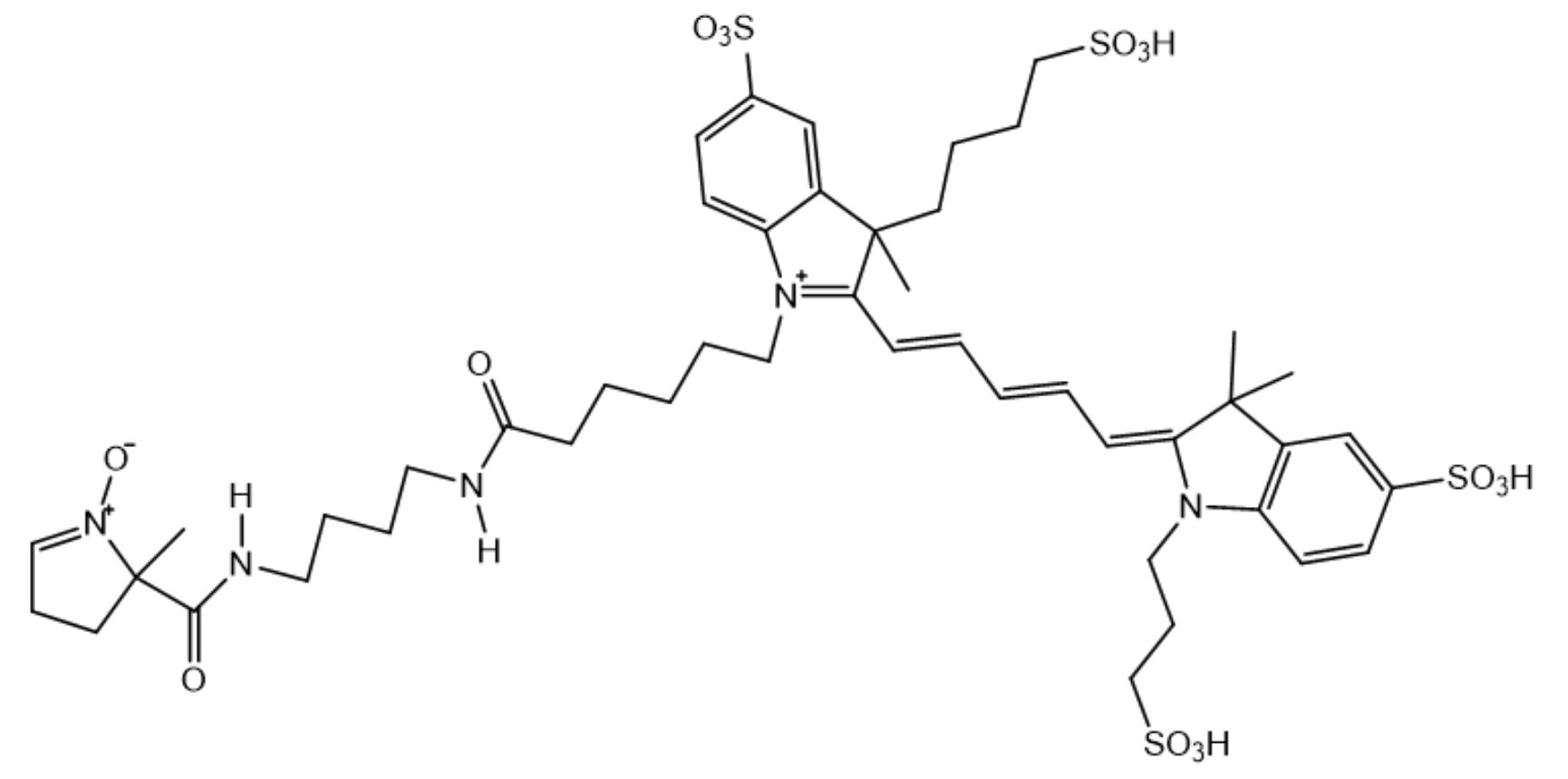


## Supplementary Figure S1.

Chemical structure of CAMPO-AFDye 647. Left segment of the structure functions serves as a spintrap, while the right part acts as a fluorophore.


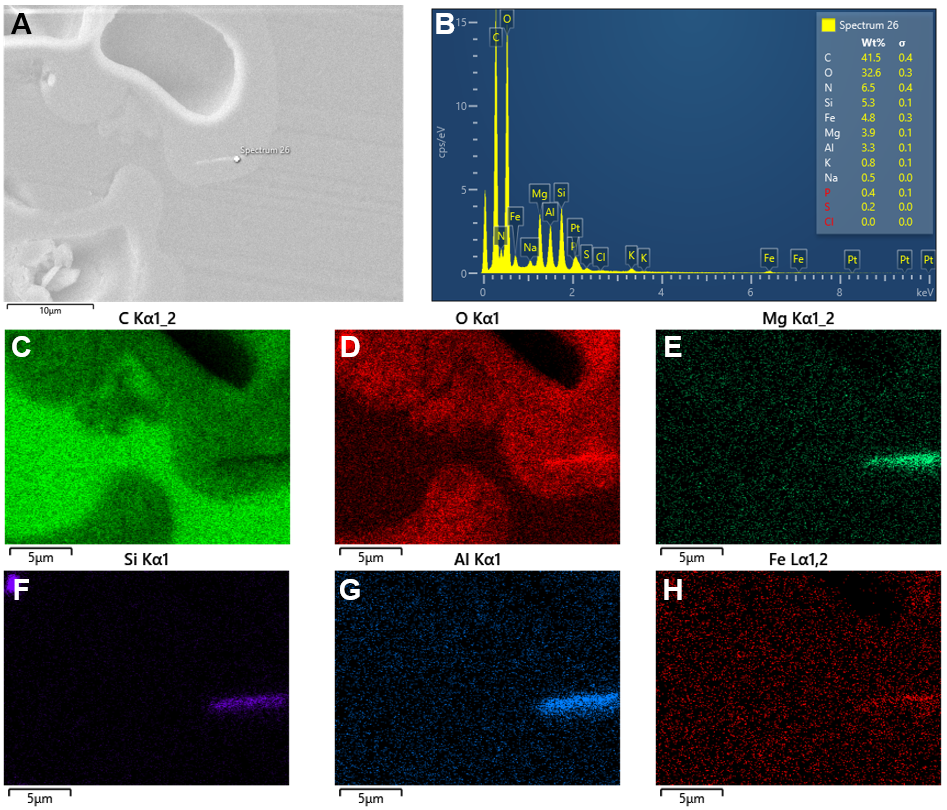


## Supplementary Figure S2.

SEM-EDX graphs of the PPI powder. A) SEM micrograph. B) Energy dispersive X-ray (EDX) spectrum. EDX Elemental mapping of C) Carbon. D) Oxygen. E) Magnesium. F) Silicium. G) Aluminum. H) Iron.

To examine the structural composition of the protein powder and identify the presence of fibers and other impurities, we studied both the protein powder itself and the protein-lipid aggregates using Scanning Electron Microscopy with Energy-Dispersive X-ray Spectroscopy (SEM-EDX). Our observations confirmed that the protein-lipid aggregates primarily consist of protein material. Additionally, it revealed the existence of contaminants, such as fibers and metallic particles, within the protein isolates. These contaminants were absent in the protein-lipid aggregates.


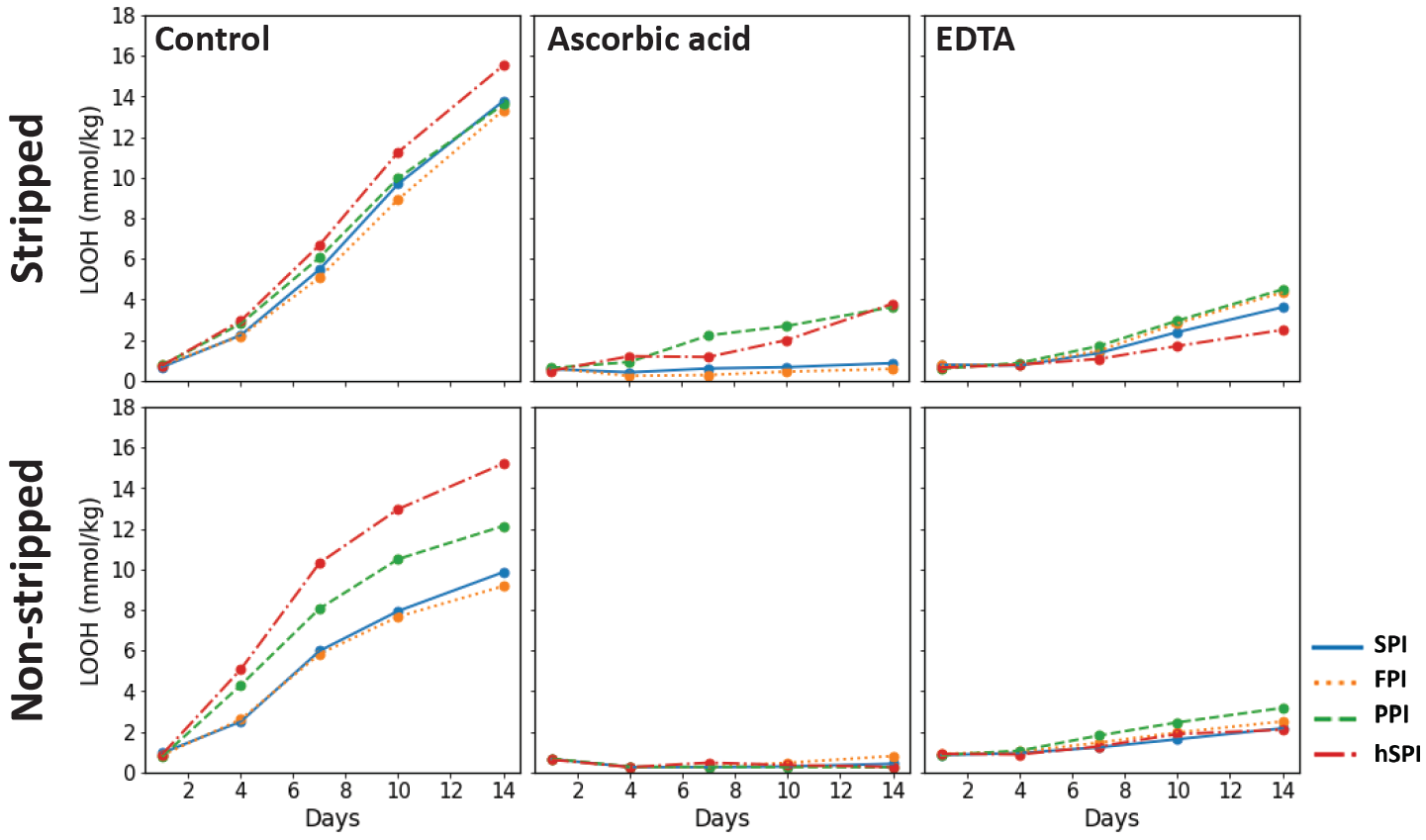


## Supplementary Figure S3.

Lipid hydroperoxide (LOOH) concentration in emulsions stored at 30°C, as determined with ^1^H NMR. Emulsions are prepared with either non-stripped or stripped oil, and with 1.8 wt% legume protein isolate (SPI, PPI, FPI, or hSPI), and contained either no supplemented antioxidant (left column), 10 mM ascorbic acid (middle column) or 1 mM EDTA (right column).


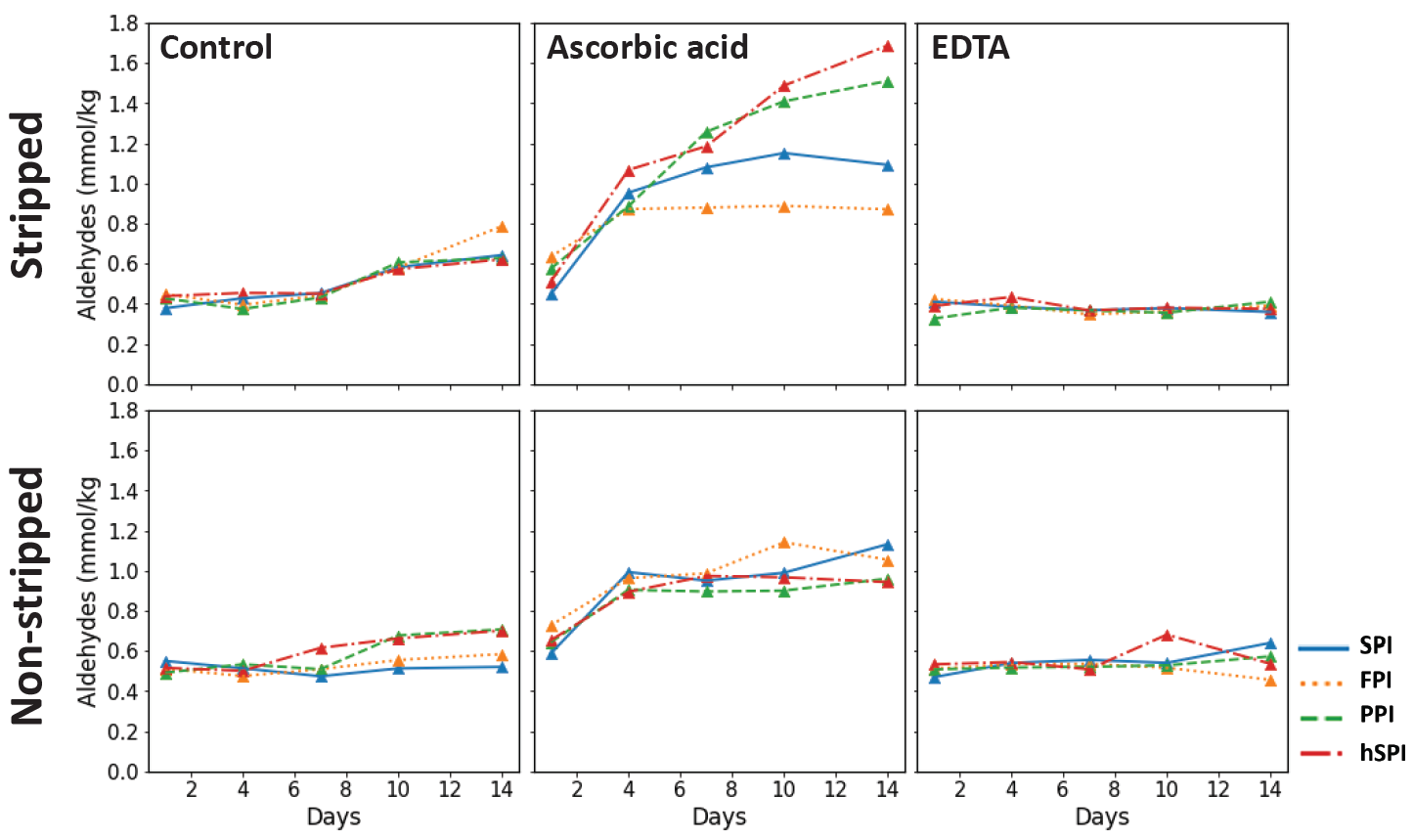


Supplementary Figure S4.

Aldehyde concentration in emulsions stored at 30°C, as determined with ^1^H NMR. Emulsions are prepared with non-stripped or stripped oil, and with 1.8 wt% SPI, PPI, FPI or hSPI, and contain either no supplemented antioxidant (control), 10 mM ascorbic acid or 1 mM EDTA.


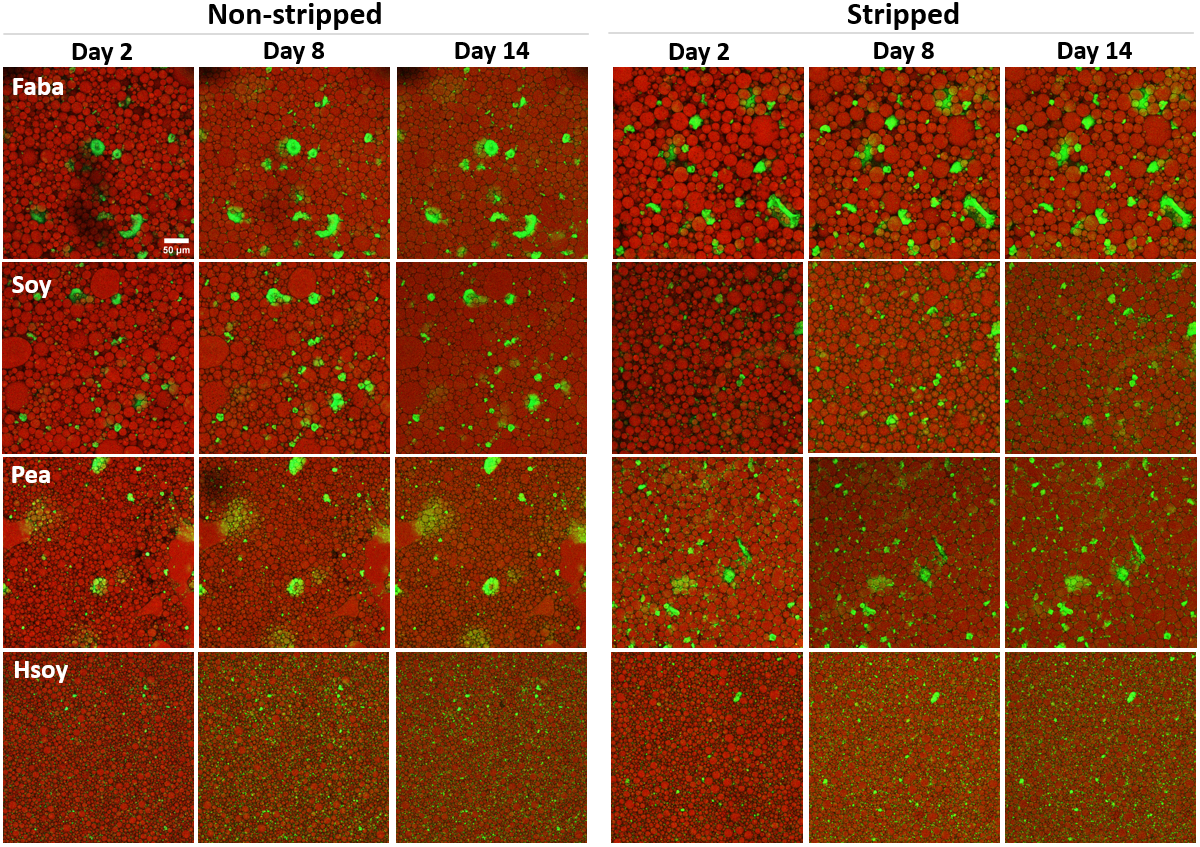


## Supplementary Figure S5.

Confocal microscopy images of non-stripped (NS) oil and stripped oil (ST) emulsions prepared with 1.8 wt% SPI, PPI, FPI or hSPI. As antioxidant, 1 mM EDTA was added to the emulsions. The emulsions were stored for 14 days at 30°C.


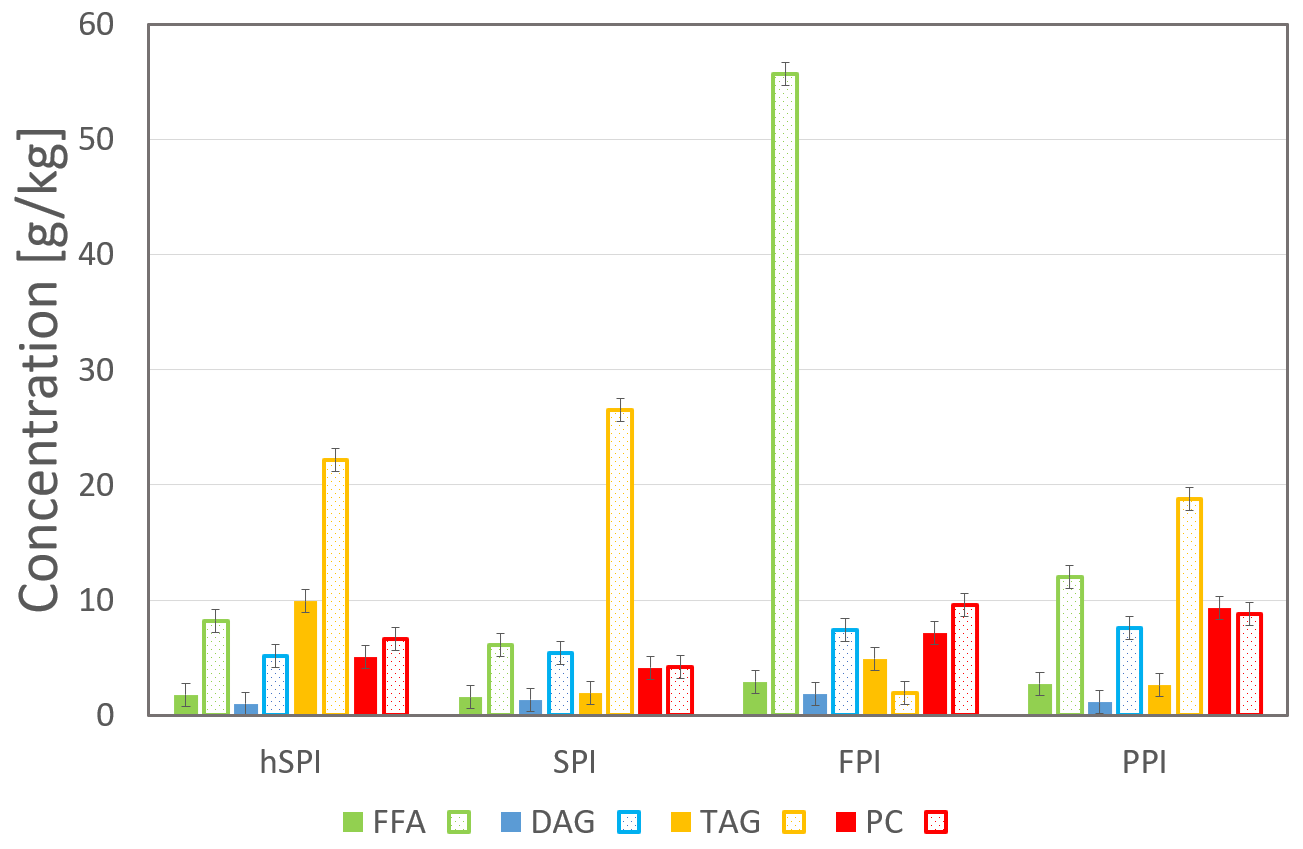


## Supplementary Figure S6.

Concentrations (based on dry weight) of free fatty acids (FFA), diacylglycerols (DAG), triacylglycerols (TAG), and phosphatidylcholine (PC) in the legume protein isolates (solid bars) and in the protein-lipid aggregates present in the emulsion water phase (open bars). The error bars are 1.0 g/kg, which is the typical error for this lipid analysis method.


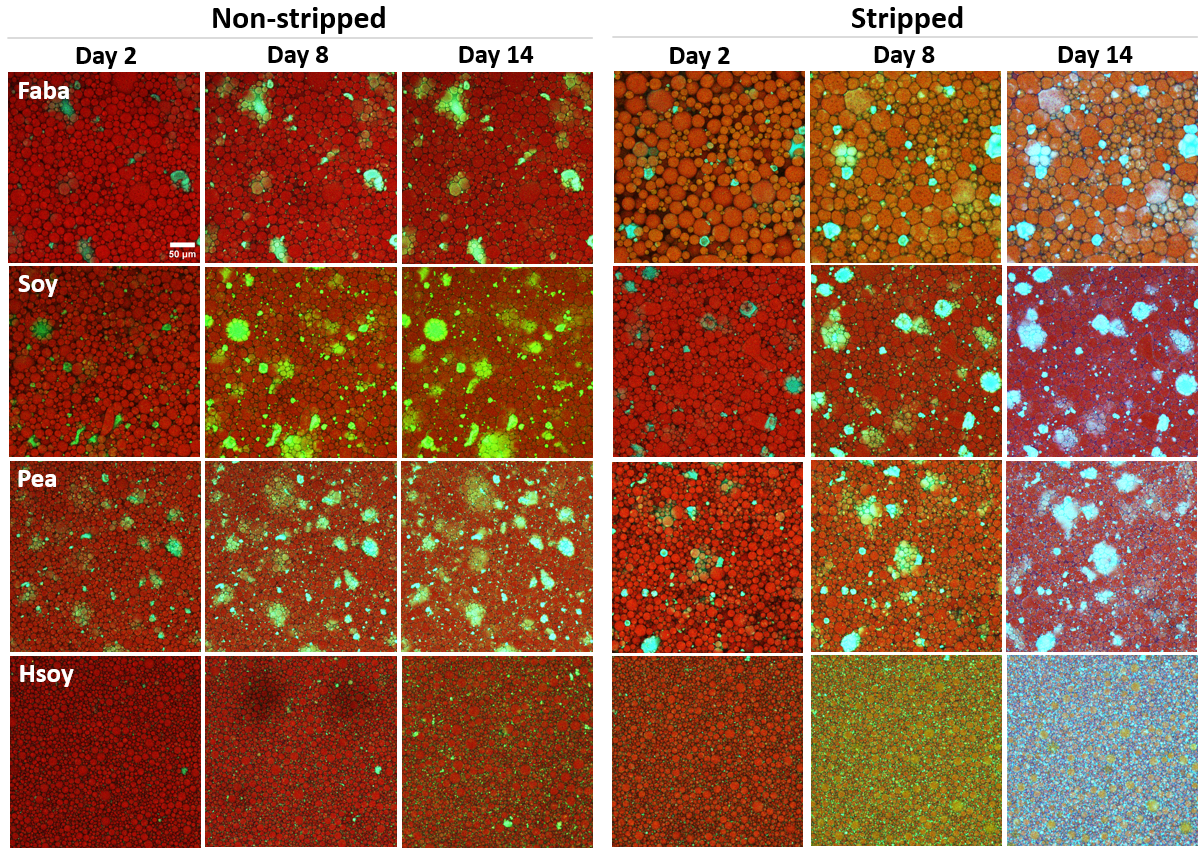


## Supplementary Figure S7.

Confocal microscopy images of non-stripped (NS) oil and stripped oil (ST) emulsions prepared with 1.8 wt% SPI, PPI, FPI or hSPI. As antioxidant, 10 mM ascorbic acid was added to the emulsions. The emulsions were stored for 14 days at 30°C.


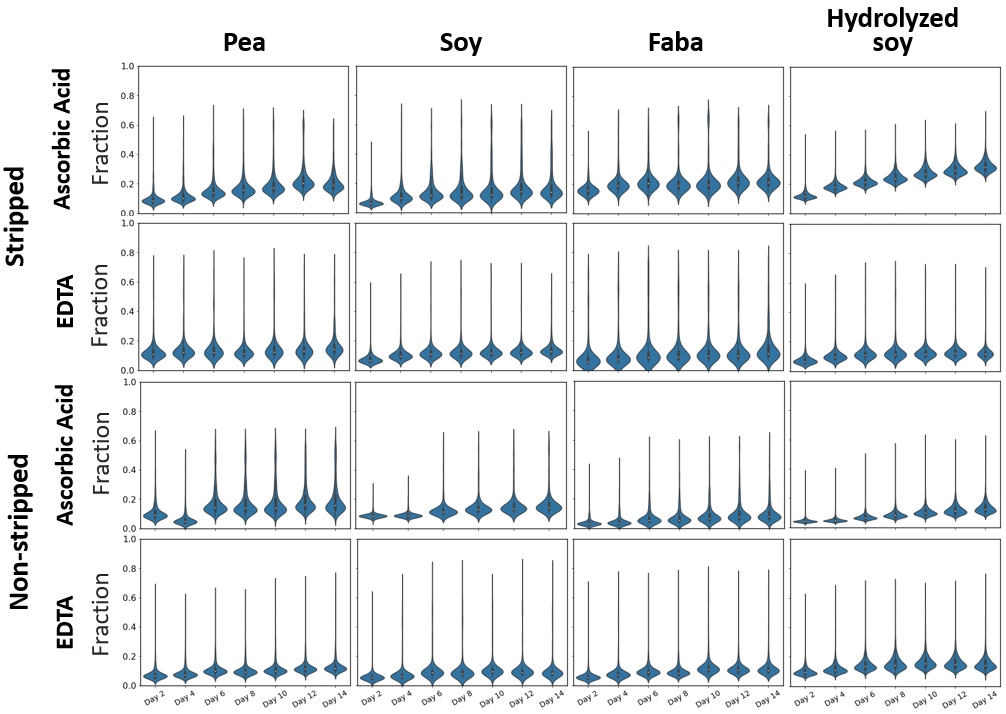


## Supplementary Figure S8.

Fraction of oxidized BODIPY 665/676 of the total BODIPY 665/676 fluorescence intensity in emulsion droplets as a function of day of storage at 30°C. Emulsions are prepared with non-stripped or stripped oil, and with 1.8 wt% SPI, PPI, FPI or hSPI, and contain either 10 mM ascorbic acid or 1 mM EDTA.


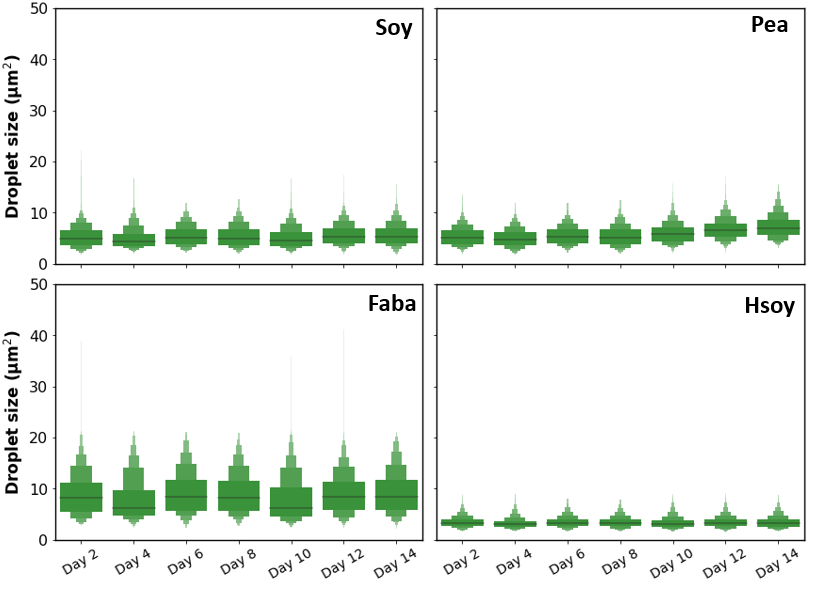


## Supplementary Figure S9.

Oil droplet size distribution in the stripped oil emulsions as function of storage time at 30°C.


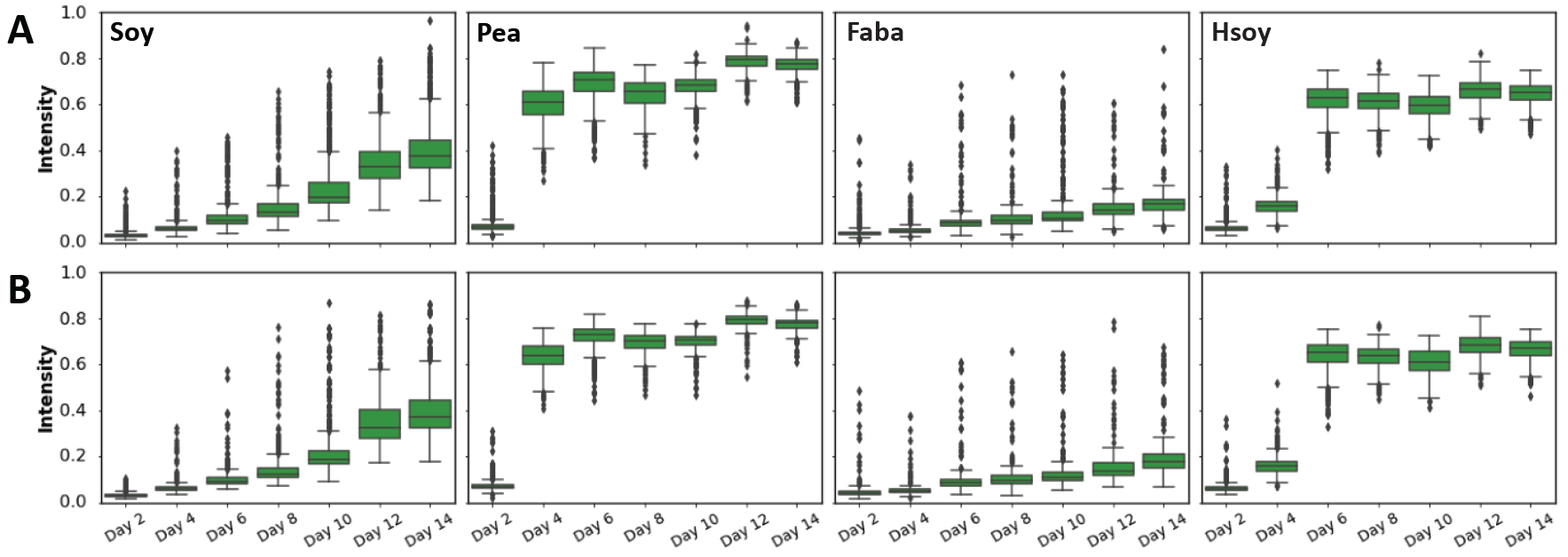


## Supplementary Figure S10.

BODIPY 665/676 fluorescence intensity at λ_ex_561 nm/λ_em_ 580-660 nm (oxidized lipids) in emulsion droplets **A)** below average droplet size and **B)** above average droplet size. Emulsions are prepared with stripped oil, and with 1.8 wt% SPI, PPI, FPI or hSPI. The emulsions were stored at 30°C for 14 days. Intensity values were normalized to the same maximum intensity value.


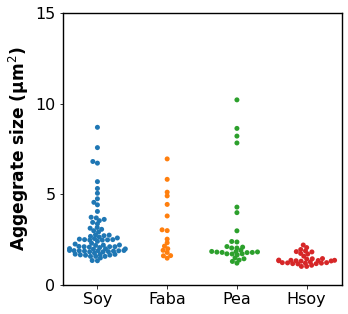


## Supplementary Figure S11.

Protein-lipid aggregate size distribution in the stripped emulsions.


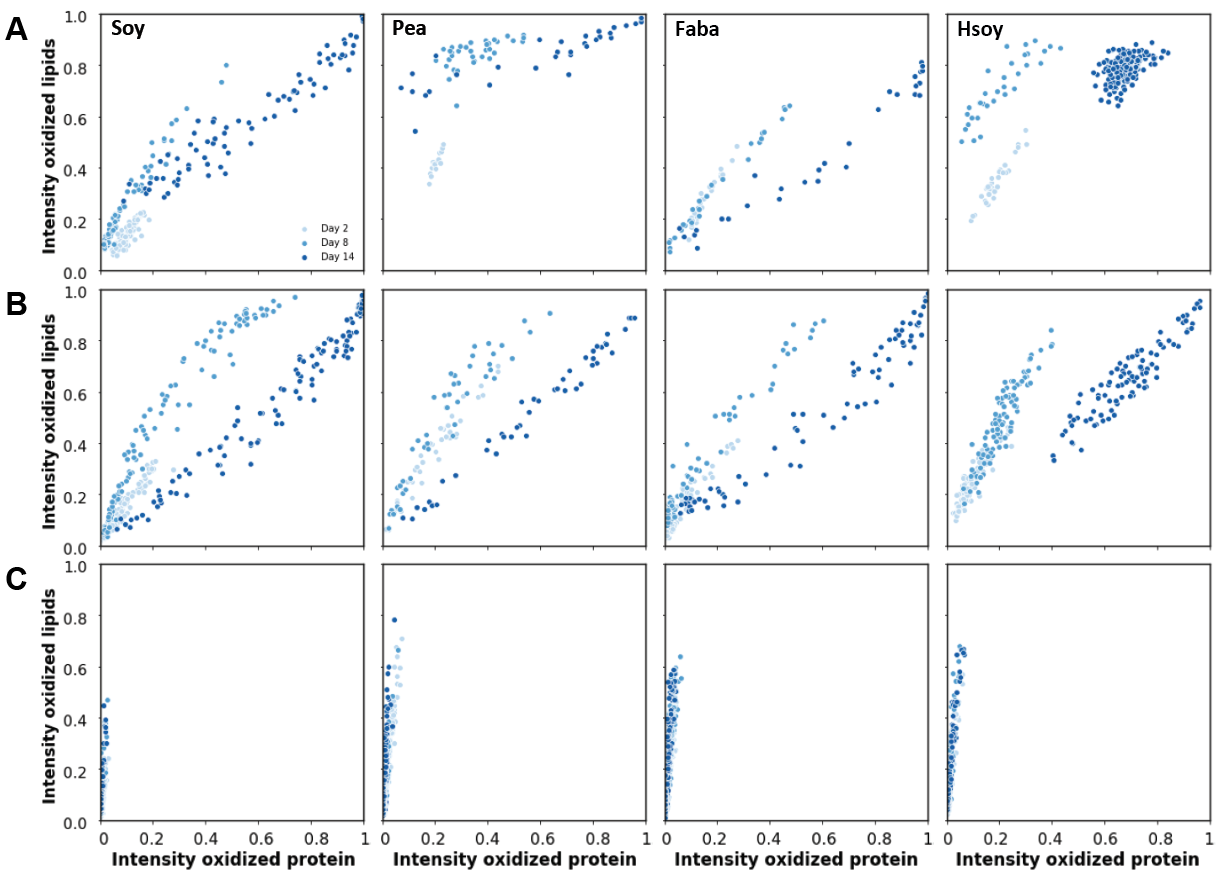


## Supplementary Figure S12.

Intensity of oxidized BODIPY 665/676 versus protein autofluorescence. Emulsions are prepared with stripped oil, and with 1.8 wt% SPI, PPI, FPI or hSPI, and contain either **A)** no supplemented antioxidant, **B)** 10 mM ascorbic acid or **C)** 1 mM EDTA. The emulsions were stored at 30°C for 14 days. Intensity values were normalized to the same maximum intensity value.


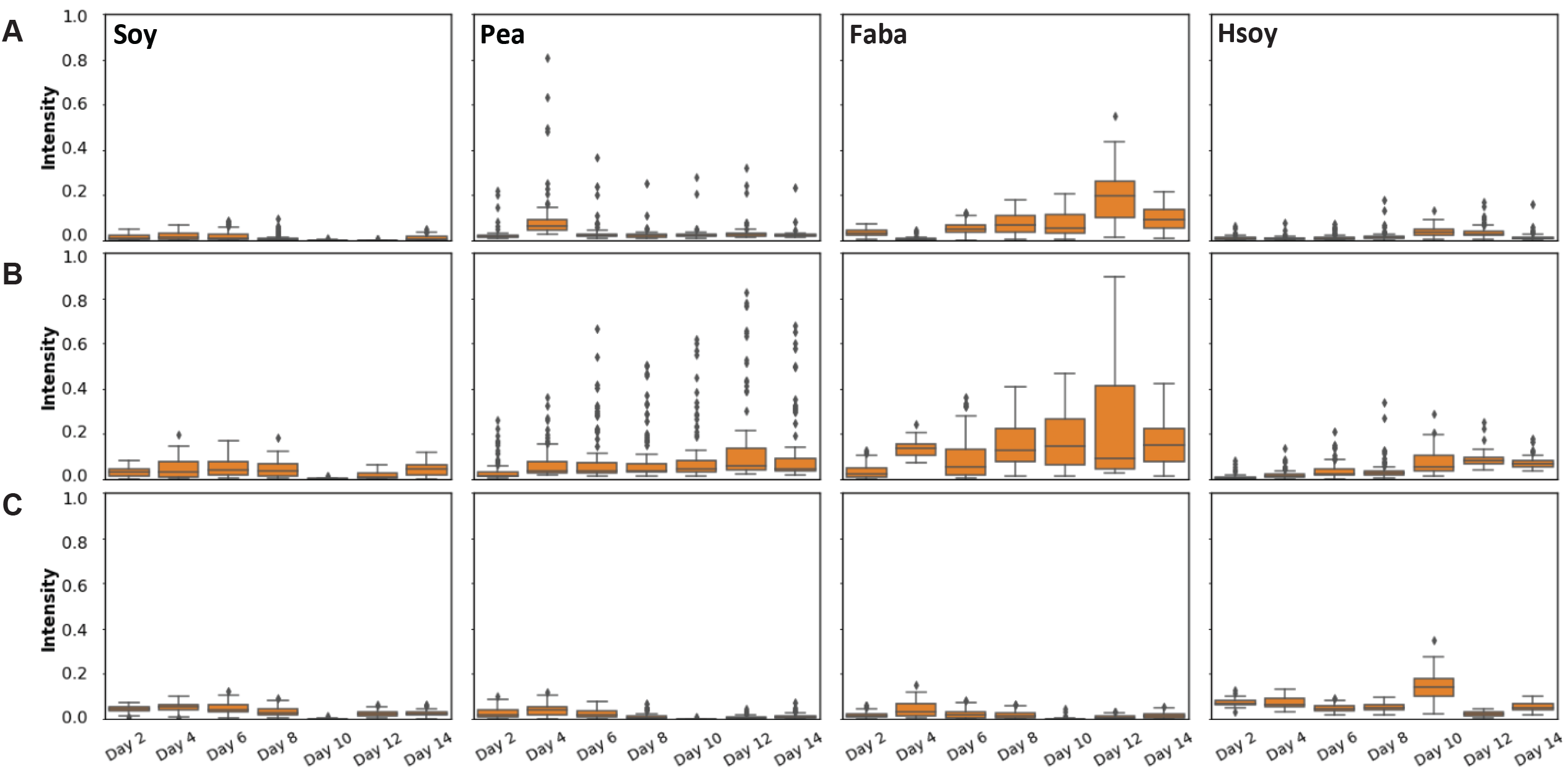


## Supplementary Figure S13.

Intensity oxidized protein in the aggregates. Emulsions are prepared with non-stripped oil, and with 1.8 wt% SPI, PPI, FPI or hSPI, and contain either **A)** no supplemented antioxidant, **B)** 10 mM ascorbic acid or **C)** 1 mM EDTA. Intensity values were normalized to the same maximum intensity value.


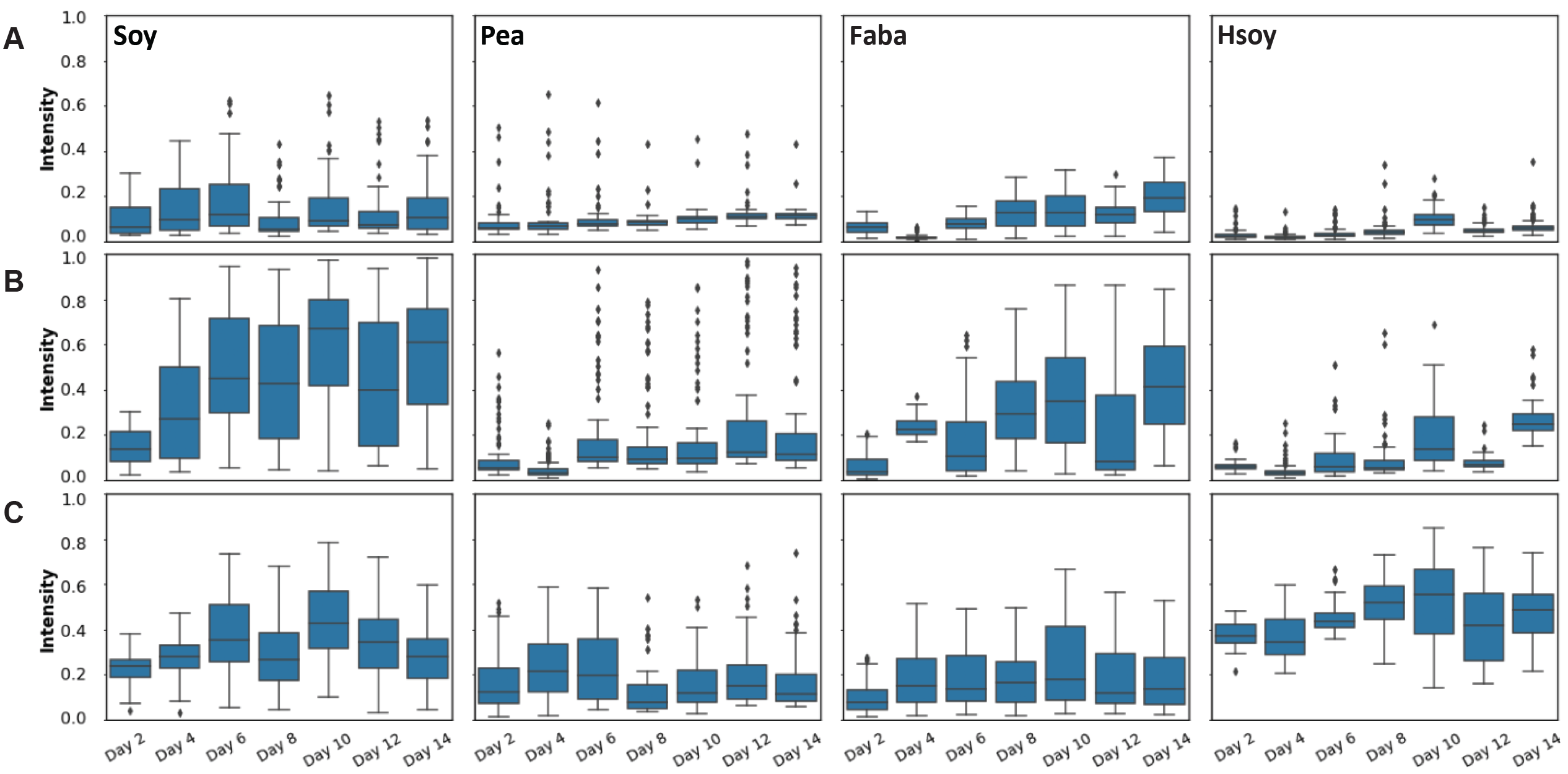


Supplementary Figure S14.

Intensity oxidized lipids in the aggregates. Emulsions are prepared with non-stripped oil, and with 1.8 wt% SPI, PPI, FPI or hSPI, and contain either **A)** no supplemented antioxidant, **B)** 10 mM ascorbic acid or **C)** 1 mM EDTA. Intensity values were normalized to the same maximum intensity value.


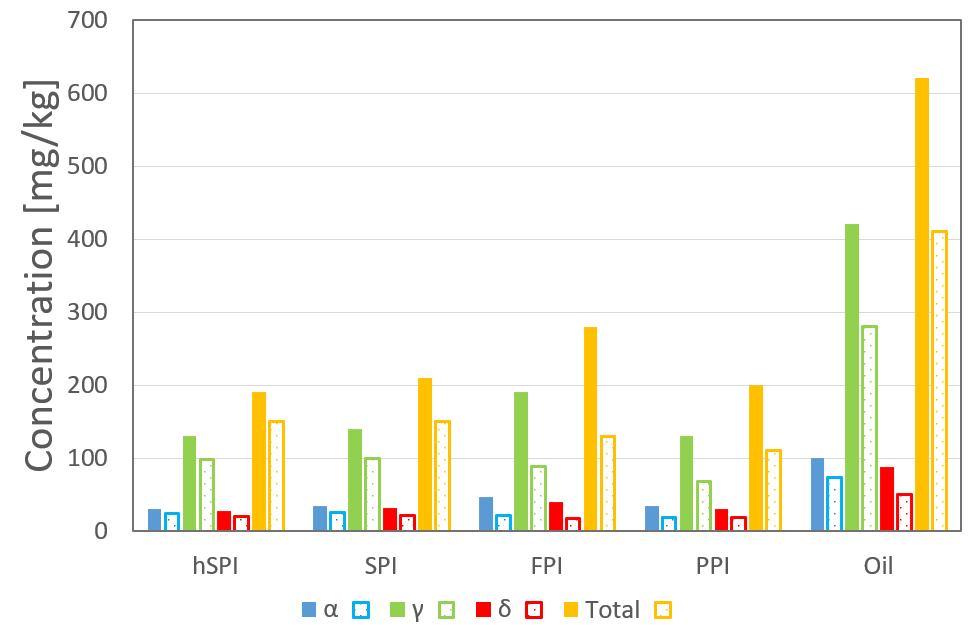


## Supplementary Figure S15.

Concentrations of α-tocopherol, γ-tocopherol, δ-tocopherol and total tocopherol concentration in the non-stripped (solid bars) and stripped oil (open bars), and in the protein-lipid aggregates present in the water phase of emulsions prepared with these oils. Note that in contrast to the other emulsions where oils were fully stripped, here the efficiency of stripping was 34%. The protein isolates used for emulsion preparation are hydrolyzed soy protein (hSPI), soy protein (SPI), faba bean protein (FPI), and pea protein (PPI). For all protein-lipid aggregates, the concentrations of β-tocopherol were below the detection limit of 10 mg/kg. In the crude protein isolates, none of the tocopherol types could be detected. The partial decrease of tocopherol in protein-lipid aggregates in the water phase corresponds the partially stripping levels in the (34%).

## Supplementary Table S1.

Additional characteristics of the legume protein isolates: (A) pH of a 1% dispersion and concentrations of phosphorus and trace minerals, (B) concentrations of phospolipids. Values are mean of duplicates.

| **A** | **pH** | **P**  **g/kg** | **Co**  **mg/kg** | **Cr**  **mg/kg** | **Mn**  **mg/kg** | **Ni**  **mg/kg** | **V**  **mg/kg** | **Zn**  **mg/kg** |
| --- | --- | --- | --- | --- | --- | --- | --- | --- |
| **FPI** | 7.69 | 10.4 | <1 | <0.5 | 13 | <1 | <0.5 | 94 |
| **PPI** | 7.62 | 9.5 | <1 | <0.5 | 8.8 | <1 | <0.5 | 102 |
| **SPI** | 6.78 | 6.6 | <1 | <0.5 | 16 | <1 | <0.5 | 30 |
| **hSPI** | 7.32 | 8.85 | <1 | <0.5 | 7.3 | <1 | <0.5 | 29 |

| **B** | **PC**  **[g/100 g]** | **PE**  **[g/100 g]** | **PI**  **[g/100 g]** |
| --- | --- | --- | --- |
| **FPI** | 1.6 | 0.6 | 0.1 |
| **PPI** | 2.4 | 0.6 | 1.5 |
| **SPI** | 1.2 | 0.4 | 1.5 |
| **hSPI** | 1.2 | 0.4 | 0.7 |

## Supplementary Table S2.

Concentrations of free fatty acids (FFA) and diacylglycerols (DAG) and triacylglycerols (TAG) in the protein-lipid aggregates present in the water phase of the emulsions. Values are based on dry weight and are the mean of duplicates.

|  | **FFA**  **g/100 g** | **DAG**  **g/100 g** | **TAG**  **g/100 g** |
| --- | --- | --- | --- |
| **SPI** | 0..6 | 0.5 | 2.6 |
| **FPI** | 0.6 | 0.7 | 0.2 |
| **PPI** | 1.2 | 0.8 | 1.9 |
| **hSPI** | 0.8 | 0.5 | 2.2 |
